# Supplementary material for: What are the consequences of caring for older people and what interventions are effective for supporting unpaid carers? A rapid review of systematic reviews
Source: BMJ Open. 2021 Sep 29;11(9):e046187. doi: 10.1136/bmjopen-2020-046187 (PMC8483048; doi:10.1136/bmjopen-2020-046187)
Supplement: Supplementary data [file bmjopen-2020-046187supp001.pdf]

Supplementary materials

Appendix A: Search strategy as applied to MEDLINE

Database(s): **Ovid MEDLINE(R)** 1946 to January Week 2 2020; Searched 21st January

#	Searches	Results
1	meta-analysis.pt.	109635
2	meta-analysis/ or systematic review/ or meta-analysis as topic/ or "meta analysis (topic)"/ or "systematic review (topic)"/ or exp technology assessment, biomedical/	205585
3	((systematic* adj3 (review* or overview* or analys*)) or (methodologic* adj3 (review* or overview* or analys*))).ti,ab,kf,kw.	150031
4	(((quantitative or narrative) adj3 (review* or overview* or synthes*)) or (research adj3 (integrati* or overview*))).ti,ab,kf,kw.	17348
5	((integrative adj3 (review* or overview*)) or (collaborative adj3 (review* or overview*)) or (pool* adj3 analy*)).ti,ab,kf,kw.	20727
6	(data synthes* or data extraction* or data abstraction*).ti,ab,kf,kw.	20899
7	(handsearch* or hand search*).ti,ab,kf,kw.	7850
8	(mantel haenszel or peto or der simonian or dersimonian or fixed effect* or latin square*).ti,ab,kf,kw.	21652
9	(met analy* or metanaly* or technology assessment* or HTA or HTAs or technology overview* or technology appraisal*).ti,ab,kf,kw.	7271
10	(meta regression* or metaregression*).ti,ab,kf,kw.	6630
11	(meta-analy* or metaanaly* or systematic review* or biomedical technology assessment* or bio-medical technology assessment*).mp,hw.	235567
12	(medline or cochrane or pubmed or medlars or embase or cinahl).ti,ab,hw.	166667
13	(cochrane or (health adj2 technology assessment) or evidence report).jw.	18482
14	(comparative adj3 (efficacy or effectiveness)).ti,ab,kf,kw.	10523
15	(outcomes research or relative effectiveness).ti,ab,kf,kw.	7615
16	((indirect or indirect treatment or mixed-treatment) adj comparison*).ti,ab,kf,kw.	1649
17	((meta-narrative or meta-ethnograph* or mixed method* or critical or thematic or realist or framework) adj3 (review* or synthes*)).ti,ab,kf,kw.	20949
18	or/1-17	395639
19	((carer* or caregiver* or caring or care partner* or "family care" or "spousal care" or "unpaid care" or "informal care") adj5 (older or senior* or elder* or dement* or alzheimer*)).ti,ab.	9395
20	18 and 19	613

Table S1. Abbreviated risk of bias criteria, adapted from the ROBIS tool[1]

1. Can searches be considered exhaustive and thus prone to minimal bias?
2. Are review criteria clear, and thus prone to minimal selection bias?
3. Is the quality assessment incorporated into the synthesis to prioritise more robust evidence, or considered in relation to the conclusions drawn?
4. Is the synthesis transparent and clear: sufficient study details are reported; synthesised evidence is referenced appropriately?
5. Do review findings and synthesis avoid emphasising results based only on statistical significance?

Table S2. The consequences of caring for older populations: summary of evidence by review risk of bias

Study	Type of consequence/impact reported					Evidence about which groups may be at greater risk
	Health (physical and mental)	Social	Finance	Quality of life	Other	
	Evidence from reviews with a moderate risk of bias					
Ge [31]	'BURDEN' (PREVALENCE)<br><br>Across 3 studies, the proportion of carers reporting levels of 'carer burden' varied from 37% to 100%.<br><br>'BURDEN' (SEVERITY)<br><br>The percentage of carers who reported high 'burden' ranged from 1% to greater than 35% (across 4 studies).	NA	NA	NA	NA	SEX<br><br>Limited evidence of an association between sex of carer (male) and higher 'carer burden'.<br><br>AGE<br><br>Younger carer age was associated with higher 'carer burden'.<br><br>SES<br><br>Limited evidence that lack of social support and insufficient financial support were factors contributing to higher 'carer burden'.
Ringer[34]	'BURDEN', DEPRESSION, ANXIETY<br><br>Carers of frail older adults experienced 'burden', depression and anxiety, but	NA	NA	NA	NA	None

	it was not possible to quantify this from included studies. Evidence from one included study indicated that the 'burden' experienced by carers of frail older people was less than that of carers of those with cancer, diabetes and dementia.					
	Evidence from reviews with a high risk of bias					
Amer [29]	DEPRESSION (SEVERITY)<br><br>Evidence about the level of depression experienced by carers differed across studies; levels were judged to be both mild to moderate, and severe, although it is not clear how these were defined.<br><br>'BURDEN' (SEVERITY)<br><br>'Carer burden' was judged to be low to moderate, although it is not clear how these are defined.	NA	NA	Carers' quality of life was lower compared to national levels.	Limited findings suggest an association between carers' perceived difficulty in assisting care recipients and differences in cultural values.	None
Bom [30]	DEPRESSION (PREVALENCE)<br><br>Caring was associated with higher prevalence of depressive feelings and lower mental health scores.<br><br>PHYSICAL HEALTH	NA	NA	NA	NA	SEX<br><br>Impact of caring on health is greater for females and those married.

	There was mixed evidence about impact on physical health; review authors suggest this reflects different outcome measures.				
Jansen [32]	DISTRESS AND ANXIETY (SEVERITY)<br><br>There was limited evidence showing greater distress and anxiety in carers of older cancer survivors compared to the general population.<br><br>SELF-ESTEEM<br><br>There was limited evidence that caring was associated with a high self-esteem.<br><br>DEPRESSION<br><br>Evidence of impact on depression was reported to be varied (although full details are not reported). Authors suggest that this was due to the variety of instruments used to measure the outcome.<br><br>'BURDEN' (PREVALENCE)		Limited evidence shows an association between lower quality of life and carers of older cancer survivors compared with the general population	There was limited evidence that carers reported difficulties in talking to the care recipients about their illness, suggesting communication issues	SEX<br><br>One study reported that being a female carer was a predictor for 'burden' while another study found that being a male spousal carer was a risk factor for 'burden'.

	Limited evidence indicated 40% of carers experienced high 'carer burden'.<br><br>STRESS (SEVERITY)<br><br>Limited evidence that carers experience higher levels of stress than the UK general adult population

Namasivayam [33]	'BURDEN' (SEVERITY)<br><br>Dysphagia in older care recipients is associated with increased 'carer burden'.	NA	NA	NA	NA

Table **S3. Interventions for carers: summary of evidence by review risk of bias**

Study	Interventions for carers of older people	
	**Findings**	**Sub-group analysis**
	Evidence from reviews with a low risk of bias	
Shaw [40]	Pooled analyses indicated respite care had no effect on 'carer burden' and anxiety, and little effect on carer depression. Some evidence indicated that respite was associated with worse carer quality of life, although the review authors note there were potential sources of bias in these studies. Other evidence indicated respite may improve the carer-recipient relationship. Home help-based respite may improve carers' morale.	None reported.
Mason [38]	There was no consistent evidence to indicate respite care was beneficial to carers. Pooled analysis indicated no effect on 'carer burden', and a small improvement in depression, although authors note a methodological flaw which undermines the reliability of this finding. However, satisfaction levels were generally high for all types of respite compared to usual care.	*Although the authors report findings from one study about outcomes for sub-groups, it is not clear if this refers to the carer or recipient. Other sub-group findings reported relate to likelihood of the carer 'institutionalising' the care recipient.*
	Evidence from reviews with a moderate risk of bias	
Murfield [39]	There was limited evidence (1 study) that a mindfulness stress reduction intervention may improve carer depression and anxiety but not other outcomes. There was limited evidence (1 study) that a yoga + meditation based intervention improved carer self-compassion and quality of life.	None reported.
	Evidence from reviews with a high risk of bias	
Domingues [35a]	There was limited and mixed evidence for a cognitive intervention and a multicomponent intervention.	None reported.
Guay [36]	There was no consistent evidence to indicate web-based education interventions were beneficial to carers, and mixed evidence for therapy interventions.	None reported.
Lopez-Hartman [37]	There was inconsistent evidence about whether individual and group psychosocial support interventions are beneficial for carers, with improvements to some outcomes but not others.	One study reported that carers sharing a household with care recipients may benefit more from an individual psychosocial support group compared to those living apart.

[a]Other interventions were reported in this review but did not target only the carer and were primarily oriented towards supporting the care recipient.

Table S4. Reviews reporting outcomes by intervention classification

	INTERVENTION TYPE				
**OUTCOME**	**Respite**	**Psychosocial**	**Cognitive**	**Education**	**Multicomponent**
**'Burden'**	Shaw 2009<br>Mason 2007	Lopez-Hartman 2012<br>Guay 2017	Domingues 2018	Guay 2017	Domingues 2018
**Depression/mood**	Shaw 2009	Lopez-Hartman 2012<br>Guay 2017	Domingues 2018	Guay 2017	Domingues 2018
**Anxiety**	Shaw 2009	Lopez-Hartman 2012<br>Guay 2017	-	-	-
**Stress, wellbeing, quality of life, 'role strain', morale, self-compassion**	Shaw 2009	Lopez-Hartman 2012<br>Murfield 2019	-	Guay 2017	
**Health, physical burden, 'other' health**	-	Guay 2017	-	-	-
**Anger, hostility**	Shaw 2009	-	-	-	-
**Knowledge**	-	Lopez-Hartman 2012<br>Guay 2017	-	-	-
**Coping**	-	Lopez-Hartman 2012<br>Guay 2017	-	-	-
**Relationships**	Shaw 2009	-	-	-	-
**Satisfaction**	Mason 2007	-	-	-	-
**Economic burden**	-	Lopez-Hartman 2012	-	-	-
