# Supplementary material for: What are the consequences of caring for older people and what interventions are effective for supporting unpaid carers? A rapid review of systematic reviews
Source: BMJ Open. 2021 Sep 29;11(9):e046187. doi: 10.1136/bmjopen-2020-046187 (PMC8483048; doi:10.1136/bmjopen-2020-046187)
Supplement: Author's manuscript [file bmjopen-2020-046187.draft_revisions.pdf]

BMJ Open is committed to open peer review. As part of this commitment we make the peer review history of every article we publish publicly available.

When an article is published we post the peer reviewers' comments and the authors' responses online. We also post the versions of the paper that were used during peer review. These are the versions that the peer review comments apply to.

The versions of the paper that follow are the versions that were submitted during the peer review process. They are not the versions of record or the final published versions. They should not be cited or distributed as the published version of this manuscript.

BMJ Open is an open access journal and the full, final, typeset and author-corrected version of record of the manuscript is available on our site with no access controls, subscription charges or pay-per-view fees (http://bmjopen.bmj.com).

If you have any questions on BMJ Open's open peer review process please email info.bmjopen@bmj.com

BMJ Open

Caring for older people: A synthesis of evidence exploring the health, social and financial consequences of caring and effective interventions for support

Journal:	*BMJ Open*
Manuscript ID	bmjopen-2020-046187
Article Type:	Original research
Date Submitted by the Author:	28-Oct-2020
Complete List of Authors:	Spiers, Gemma; Newcastle University, Population & Health Sciences Institute<br>Liddle, Jennifer; Newcastle University, Population & Health Sciences Institute; Newcastle University, Population & Health Sciences Institute<br>Kunonga, Tafadzwa; Newcastle University, Population & Health Sciences Institute<br>Whitehead, Orla; Newcastle University, Population Health Sciences Institute<br>Beyer, Fiona ; Newcastle University, Population & Health Sciences Institute<br>Stow, Daniel; University of Newcastle upon Tyne, Population and Health Sciences Institute; Newcastle University<br>Welsh, Claire; Newcastle University, Population & Health Sciences Institute<br>Ramsay, Sheena; Newcastle University, Population & Health Sciences Institute<br>Craig, Dawn; Newcastle University, Health Economics Group, Population & Health Sciences Institute<br>Hanratty, Barbara; Newcastle University, Population & Health Sciences Institute
Keywords:	Health policy < HEALTH SERVICES ADMINISTRATION & MANAGEMENT, GERIATRIC MEDICINE, PRIMARY CARE

SCHOLARONE™
Manuscripts

I, the Submitting Author has the right to grant and does grant on behalf of all authors of the Work (as defined in the below author licence), an exclusive licence and/or a non-exclusive licence for contributions from authors who are: i) UK Crown employees; ii) where BMJ has agreed a CC-BY licence shall apply, and/or iii) in accordance with the terms applicable for US Federal Government officers or employees acting as part of their official duties; on a worldwide, perpetual, irrevocable, royalty-free basis to BMJ Publishing Group Ltd ("BMJ") its licensees and where the relevant Journal is co-owned by BMJ to the co-owners of the Journal, to publish the Work in this journal and any other BMJ products and to exploit all rights, as set out in our licence.

The Submitting Author accepts and understands that any supply made under these terms is made by BMJ to the Submitting Author unless you are acting as an employee on behalf of your employer or a postgraduate student of an affiliated institution which is paying any applicable article publishing charge ("APC") for Open Access articles. Where the Submitting Author wishes to make the Work available on an Open Access basis (and intends to pay the relevant APC), the terms of reuse of such Open Access shall be governed by a Creative Commons licence – details of these licences and which Creative Commons licence will apply to this Work are set out in our licence referred to above.

Other than as permitted in any relevant BMJ Author's Self Archiving Policies, I confirm this Work has not been accepted for publication elsewhere, is not being considered for publication elsewhere and does not duplicate material already published. I confirm all authors consent to publication of this Work and authorise the granting of this licence.

For peer review only

Caring for older people: A synthesis of evidence exploring the health, social and financial consequences of caring and effective interventions for support

Spiers, Gemma [a] * gemma-frances.spiers@newcastle.ac.uk

Liddle, Jennifer [a, b]

Kunonga, Tafadzwa Patience [a]

Whitehead, Orla Ishbel [a]

Beyer, Fiona [c]

Stow, Daniel [a]

Welsh, Claire [a]

Ramsay, Sheena [c]

Craig, Dawn [c]

Hanratty, Barbara [a]

[a] Population and Health Sciences Institute, Newcastle University, Campus for Ageing & Vitality, Newcastle upon Tyne, NE4 5PL
[b] Open Lab, Newcastle University, Newcastle Helix, Newcastle upon Tyne, NE4 5TG
[c] Population and Health Sciences Institute, Newcastle University, Baddiley-Clark, Newcastle upon Tyne, NE2 4AX

*Corresponding author

Word count: 3485
References: 53
Tables: 3 (plus four in supplementary materials)
Figures: 2

Funding statement

This paper presents independent research funded by Public Health England (NU-002487 RES/0290/0170). The views expressed are those of the author(s) and not necessarily those of Public Health England.

Author contributions

GS developed the protocol, undertook all stages of the review and co-wrote the first draft of the paper; JL developed the protocol, undertook all stages of the review and co-wrote the first draft of the paper; PK developed the protocol, undertook all stages of the review and contributed to the final draft of the paper; OW developed the protocol, undertook record

For peer review only
For peer review only - http://bmjopen.bmj.com/site/about/guidelines.xhtml

screening and data extraction for the review and contributed to the final draft of the paper; FB developed the protocol, undertook the study searches, and contributed to the final draft of the paper; DS developed the protocol, undertook record screening for the review and contributed to the final draft of the paper; CW developed the protocol, undertook record screening for the review and contributed to the final draft of the paper; SR developed the protocol, undertook record screening for the review and contributed to the final draft of the paper; DC planned the study, developed the protocol, undertook record screening for the review and co-wrote the first draft of the paper; BH planned the study, developed the protocol, undertook record screening and co-wrote the first draft of the paper.

For peer review only

For peer review only - http://bmjopen.bmj.com/site/about/guidelines.xhtml

Caring for our older people: A synthesis of high-level evidence exploring the health, social and financial consequences of caring and effective interventions for support

Objectives: To identify and map evidence about the consequences of unpaid caring for all carers of older people, and effective interventions to support this carer population.

Design: A rapid review of systematic reviews, focused on the consequences for carers of unpaid caring for older people, and interventions to support this heterogeneous group of carers. Reviews of carers of all ages were eligible, with any outcome measures relating to carers' health, social and financial wellbeing. Searches were conducted in MEDLINE, PsycInfo and Epistemonikos (January 2000 - January 2020). Records were screened, and included systematic reviews were quality appraised. Summary data were extracted and a narrative synthesis produced.

Results: Twelve systematic reviews reporting evidence about the consequences of caring for carers (n=6) and assessing the effectiveness of carer interventions (n=6) were included. The review evidence typically focused on mental health outcomes, with little information identified about carers' physical, social and financial wellbeing. Clear estimates of the prevalence and severity of carer outcomes, and how these differ between carers and non-carers, were absent. A range of interventions were identified, but there was no strong evidence of effectiveness. In some studies, the choice of outcome measure may underestimate the full impact an intervention.

Conclusions:

Current evidence fails to fully quantify the impacts that caring for older people has on carers' health and wellbeing. Information on social patterning of the consequences of caring is absent. Systematic measurement of a broad range of outcomes, with comparison to the general population, is needed to better understand the true consequences of caring. Classification of unpaid caring as a social determinant of health could be an effective lever to bring greater focus and support to this population. Further work is needed to develop and identify suitable interventions, in order to support evidence-based policymaking and practice.

For peer review only - http://bmjopen.bmj.com/site/about/guidelines.xhtml

Strengths and limitations of this study

- A rapid review of published systematic reviews offers a useful and efficient approach to summarising evidence about caring for older people, without duplicating existing work.
- This approach allowed us to identify key gaps in this evidence base and make clear recommendations for future research.
- A limitation of this method is the exclusion of primary research studies that have not yet been subject to a systematic review.
- Most of the included systematic reviews were published within the last three years, signalling a contemporary evidence base.

For peer review only

For peer review only - http://bmjopen.bmj.com/site/about/guidelines.xhtml

Background

People aged 85 years and over belong to the fastest growing age group in the UK, and form a population that is expected to double to 3.2 million by 2041.[1] The growth of this population will inevitably result in an increase in care needs. Indeed, current projections suggest that the number of dependent older people in the UK will increase by 113% by 2051.[2] Current state provision of social care has failed to keep pace with current levels of demand.[3 4] For example, around 1.5 million older people are estimated to have an unmet need for care.[5] The consequences of this gap between care needs and care provision are also likely to be experienced by unpaid carers. Indeed, there are approximately 6.5 million people in the UK providing unpaid care to ill and disabled family members, friends or partners,[6] with an estimated economic contribution of up to £132 billion per year.[7]

Unpaid carers make a critical contribution to health and social care.[8] Yet caring can have profound impacts on the lives of carers. The demands of caring bring emotional, health and financial challenges,[9 10] alongside changes to home and daily routines, work and career, family and social networks.[11-13] Carers are at greater risk of premature death and higher disease prevalence, whilst neglect of their own health care needs is common.[14] Such poor health is likely to be exacerbated by the social isolation, poor information and support, and financial stress that carers experience.[15] Supporting the mental and physical health and wellbeing of carers is essential. It is also critical that any form of support must account for the complexity of caring. For example, six key attributes of the caring experience (for those caring for an older person) have been identified as: getting on with the care recipient; assistance from organisations and the government; support from family and friends; activities outside caring; control; and fulfilment.[16] These attributes reflect the need to consider a broad range of outcomes when designing, implementing and evaluating carer support interventions.

Recognition of the role and contribution of carers has grown since the 1960s.[17] In the UK, National Institute for Health and Care Excellence (NICE) guidelines for supporting carers were published in 2020, highlighting this population as a policy priority.[18]

For peer review only - http://bmjopen.bmj.com/site/about/guidelines.xhtml

Evidence informed practice will ensure that the most effective interventions are used to support carers, whilst targeting the most relevant outcomes. As the number of older people requiring care rises, it is important that evidence is relevant to the contribution and support needs of people caring for older populations. Recent umbrella reviews have focused on evidence on interventions for carers more broadly or carers of people with dementia.[19-22] Whilst this is important, there is now a clear need to map the evidence about carers of older people, with a focus on both the consequences of caring *and* the most effective forms of carer support. This will enhance our understanding of how best to support carers of older people.

We under undertook a review of systematic reviews to map current evidence about caring for an older person, addressing two key questions:

1. What are the consequences of caring for older people for the health, social and financial wellbeing of carers, and what do we know about how these consequences vary by age, sex, socioeconomic status and geographical location?

2. Which interventions are effective (including consideration of costs) to promote health and wellbeing and access to services amongst these carers?

The aim of this work was to provide an overview of what is known, as well as identifying key gaps, to support evidence informed practice for those caring for older people.

Methods

The approach to this evidence synthesis was a rapid review of published systematic reviews (from here on referred to as 'rapid review'). Preliminary scoping of the literature identified multiple systematic reviews on both the consequences of caring and associated interventions to support those caring for older people. Thus, the review of reviews ('umbrella review') approach was most appropriate as an efficient approach to assessing the evidence, without duplicating existing research. Rapid

For peer review only - http://bmjopen.bmj.com/site/about/guidelines.xhtml

review methodology was employed, which uses a streamlined approach to study selection and synthesis in order to produce a timely overview of evidence.[23 24]

The following methods are reported in accordance with Preferred Reporting Items for Systematic Reviews and Meta-Analyses (PRISMA) guidelines.

Search strategy

A search strategy was developed, piloted and refined, combining terms for 'carers' with validated systematic review filters (see supplementary materials).[25] Searches were carried out in MEDLINE, PsycInfo and Epistemonikos (https://www.epistemonikos.org/) in January 2020, and were limited to English language publications published after 2000.

Review criteria

Table 1 outlines the review criteria. Eligible systematic reviews were those that: reported evidence about the consequences of caring, or the effectiveness of interventions for promoting carer health and wellbeing and access to services, where the care recipients were older people. Initial scoping indicated that many reviews *did not* specify an age threshold (e.g. >65 years) for older care recipients. Therefore, no age criteria for care recipients were specified in this rapid review. Instead, reviews were included if the care recipient population was described as older people, or where the recipient population was likely to include older populations. In consultation with a study steering group, reviews that focused solely on carers of people with dementia were excluded, as evidence about dementia caregivers has been summarized in three recent reviews.[19-21] All carer populations were eligible with no limits on age and sex.

As the focus of the (second) review question was about interventions to promote *carers'* health, wellbeing and access to services, reviews of *joint carer and recipient* interventions were excluded. Eligible outcomes were any that related to carers' health, social and financial wellbeing, and access to services.

For peer review only - http://bmjopen.bmj.com/site/about/guidelines.xhtml

Reviews published after 2000 in English were included, and reviews of studies with any study design were eligible. Reviews must have met at least three of the five Database of Abstracts of Reviews of Effects (DARE) criteria to be considered a systematic review, with criterion 3 being mandatory.[26] Four out of five DARE criteria is the usual threshold to be considered a systematic review. However, this was lowered to three criteria here, to maximise the number of reviews captured and thus evidence scoped. Criterion 3 was mandatory, as quality assessment of included studies is a key component of identifying the most robust evidence.

For peer review only

For peer review only - http://bmjopen.bmj.com/site/about/guidelines.xhtml

Table 1. Review criteria

	INCLUSION CRITERIA	
	**SYNTHESIS 1: CONSEQUENCES OF CARING**	**SYNTHESIS 2: INTERVENTIONS FOR CARERS**
**POPULATION**	Carers (e.g. unpaid, family, 'informal') of older adults. No age criteria for care recipients is specified, but must be, or likely to include, older populations (e.g. people with dementia).<br><br>Carers include people of all ages, male and female.<br><br>Care recipients (i.e. older adults) include male and female, with any medical diagnosis, impairment, disability, or frailty, and no limits to ethnicity.<br><br>Setting – Care provided in the community, hospital, care home.	
**INTERVENTION**	*Not applicable*	Any carer intervention that is targeted only on the carer (i.e. not a joint carer and care recipient intervention) and which aims to improve carers' health, wellbeing and/or access to services.
**COMPARATOR**	No comparator, or non-carers	Any or no comparator, including usual care
**OUTCOME**	Health status, quality of life, well-being, incident ill-health, admission to hospital, financial wellbeing, poverty, measured changes in material circumstances, social relationships including loneliness, isolation, social support, social networks.	
**STUDY DESIGN**	Systematic reviews (those that meet 3 of 5 DARE criteria). Publication dates 2000-2019. If more recently published systematic reviews include evidence that is also in earlier reviews, the most recent reviews will be prioritised to avoid duplication.<br><br>English language publications	

For peer review only - http://bmjopen.bmj.com/site/about/guidelines.xhtml

Study selection

Records were managed in Rayyan software (https://rayyan.qcri.org), an online platform to manage and assist screening for systematic reviews.[27] Titles and abstracts were screened for relevance: 20% by two researchers independently, and the remaining 80% by a single researcher, as per rapid review methods.[23] For the 20% of records screened jointly, disagreements were resolved by taking forward any records, for which decisions differed, to the next stage of screening. Records that were deemed relevant based on title and abstract were retrieved for full text screening against the review criteria by one researcher.

Data extraction

Separate data extraction forms were developed and piloted for each review question, using an Excel spreadsheet. Review details and characteristics were extracted, including: author, year of publication, number of studies included in the review, carer and care recipient population, type of consequence reported, interventions evaluated, outcomes, and type of synthesis. Review findings and conclusions were summarised, including any sub-group analyses where reported.

Quality appraisal

A full risk of bias assessment is not typically part of a rapid review.[23 24] However, an *indication* of bias was deemed important for this synthesis in order to identify the most robust findings, and obtain an overview of the quality of the evidence base. Thus, an abbreviated risk of bias appraisal was completed for included reviews using an amended version of the Risk of Bias in Systematic Reviews (ROBIS) tool.[28]

This appraisal used five items relating to review methodology that indicate likely risk of bias (supplementary materials table S1). Each item was scored 1 for 'yes' and the total score was summed across the five items. A score of 5 indicates a low risk of bias; 4 indicates a moderate risk of bias; three or less indicates a high risk of bias.

For peer review only - http://bmjopen.bmj.com/site/about/guidelines.xhtml

Data synthesis

A narrative synthesis was used to address each question, with reviews grouped into those reporting caring consequences (question 1) and those reporting effectiveness of carer interventions (question 2). Summary tables were produced to describe review characteristics, the type of evidence identified, and indicative risk of bias. Findings were synthesised: first by the judged risk of bias (i.e. low, moderate and high risk of bias) to prioritise the most robust evidence; and then by the type of consequence (question 1) and type of intervention (question 2). For review question 2, all identified interventions from included reviews were mapped against the outcomes reported to identify key gaps in evidence.

Patient and public involvement

A steering group representing diverse perspectives and organisations provided advice on the content of the review and dissemination, in regular meetings. In particular, we excluded carers of people with dementia from this study after discussion with them.

Findings

Twelve systematic reviews met the review criteria (Figure 1). Six reviews reported evidence about the consequences of caring (Table 2)[29-34] and six reported evidence about carer interventions (Table 3).[35-40] In this section, we present the synthesis of evidence about the consequences of caring for older people (question 1) and the effectiveness of carer interventions (question 2). Evidence is reported according to judged risk of bias to prioritise the most robust findings.

For peer review only - http://bmjopen.bmj.com/site/about/guidelines.xhtml

Table 2. Overview of systematic reviews reporting evidence about the consequences of caring

Study	Date	Number of studies in review	Data range of included studies	Population (care recipient)	Type of consequence/impact reported	Do studies within the review compare presence/level of impact with non-carer samples?	Does the review identify evidence about impact for specific carer sub-groups relating to age, sex, socioeconomic status and geographical location?	Synthesis	Indicative risk of bias
Amer[29]	2019	19	1990-2017	Older people	'Caregiver burden', quality of life, perceived difficulty assisting recipient	No	No	Narrative	HIGH
Bom [30]	2019	15	2009-2017	Older people	Mental health, physical health	Yes, controls matched for caregiver health	Sex, SES, location (European region)	Narrative	HIGH
Ge [31]	2017	7	1999-2014	Older people with cancer	'Caregiver burden' prevalence and severity	No	Sex, SES, age	Narrative	MODERATE
Jansen [32]	2018	14	1994-2016	Older cancer survivors	Prevalence and predictors of 'caregiver burden', depression, anxiety, self-esteem, distress, communication issues, stress and QoL	Yes, general population	Sex, SES	Narrative	HIGH
Namasivayam [33]	2018	4	2002-2017	Older people	'Caregiver burden' associated with feeding difficulties	No	No	Narrative	HIGH
Ringer [34]	2017	9	1997-2012	Older people with frailty	'Caregiver burden', negative reactions to caregiving	Unclear	No	Narrative	MODERATE

For peer review only - http://bmjopen.bmj.com/site/about/guidelines.xhtml

Table 3. Overview of systematic reviews reporting evidence about carer interventions

Study	Date published	Number of studies in review	Data range of included studies	Population (care recipient)	Interventions evaluated	Outcome	Synthesis	Indicative risk of bias
Domingues[35]	2018	4	2013-2016	Older people with mild cognitive impairment	Cognitive, multicomponent	'Burden', quality of life, anxiety, mood	Narrative	HIGH
Guay[36]	2017	12	2000-2015	Older people	Internet-based interventions, including education, self-help therapeutic, and human-supported therapeutic.	Depression, anxiety, stress, 'strain', 'burden', physical burden, self-perceived health, quality of life	Narrative	HIGH
Lopez-Hartman[37]	2012	10	2002-2009	Older people	Individual and group psychosocial support	Depression, 'burden', stress, 'role strain', coping, knowledge, social support, anxiety, economic burden.	Narrative	HIGH
Mason[38]	2007	22	1985-2003	Older people	Respite	Satisfaction, 'burden'	Both	LOW
Murfield[39]	2019	4	2013-2018	Older people	Mindfulness + stress reduction, yoga and meditation.	Self-compassion, 'other health' outcome	Narrative	MODERATE
Shaw[40]	2009	104	2005-2009	Older people	Respite	'Burden', depression, anxiety, morale, anger, hostility, caregiving relationship	Both	LOW

For peer review only - http://bmjopen.bmj.com/site/about/guidelines.xhtml

Figure 1 here

Consequences of caring

Of the six systematic reviews reporting evidence about the consequences of caring for older people: none were judged to have a low risk of bias; two a moderate risk of bias;[31 34] and four a high risk of bias[29 30 32 33] (Supplementary materials table S2).

Moderate risk of bias

Two reviews reported that carers of older people experience 'burden'[a], depression and anxiety, but prevalence and severity were either not quantified, or estimates varied substantially.[31 34] For example, one review reported the prevalence of 'burden' among carers ranged from 1% to greater than 35%,[34] whilst the other reported estimates ranging from 37% to 100%.[31] The latter review presented limited evidence on which groups may be at greatest risk - this suggested that carers who are younger, male and with poor social and financial support may experience higher levels of 'burden'.[31] However, this evidence was not quantified in the review and so it is not possible to describe the difference in reported 'burden' between these groups.

High risk of bias

Four reviews reported that carers of older people experience 'burden', anxiety and stress, but prevalence and severity were either not quantified or highly variable.[29 30 32 33] Evidence about the severity of depression among carers was also variable.[29 30 32] For example, one review reported evidence of mild, moderate *and* severe depression, although it was not clear how 'mild', 'moderate' and 'severe' were defined.[29] Another review reported that the impact on depression varied, but with no supporting detail.[32] One review indicated that levels of carer stress, anxiety and

[a] Due to insufficient detail reported in publications about the outcomes 'carer burden' and 'role strain', we are unable to provide a clear definition of these outcomes.

For peer review only - http://bmjopen.bmj.com/site/about/guidelines.xhtml

distress were higher than those of the general population, although by how much was not reported.[32]

There was limited and mixed evidence about the consequences of caring for physical health in one review, with a positive impact on self-rated health but also evidence of increased pain and medication usage.[30] Evidence about which groups may be at greatest risk was reported in two reviews.[30 32] One suggested that the impact of caring on health was greater for females and married people.[30] Yet another reported mixed evidence about whether 'burden' was greater for male or female carers.[32]

Carer interventions

Of the six reviews reporting evidence about interventions to support those caring for older people, two were judged to have a low risk of bias;[38 40] one a moderate risk of bias;[39] and three a high risk of bias[35-37] (Supplementary materials table S3).

Low risk of bias

Two reviews reported evidence on respite interventions for carers of older people.[38 40] Carers valued respite, with high levels of reported satisfaction. Yet there was no strong evidence to suggest respite improved carers' mental health or 'carer burden.' There were gaps in the evidence about the impact of respite on many other types of outcomes (e.g. physical health).

Moderate rate of bias

Limited evidence was presented in one review, for the ability of a mindfulness stress reduction intervention to improve carer depression and anxiety. However, this intervention had no impact on stress, health service use, quality of life, or self-compassion. In the same study, a combined yoga and meditation intervention appeared to improve carer self-compassion and quality of life.[39]

High risk of bias

For peer review only - http://bmjopen.bmj.com/site/about/guidelines.xhtml

Reviews judged to have a high risk of bias reported evidence about cognitive-, psychosocial- and education-based interventions.

A review judged to be at high risk of bias, reported limited and inconsistent evidence that a cognitive-based intervention was beneficial to carers.[35] The components of this cognitive intervention included 'calendar training' and 'note taking', although the overall objective of this intervention was not clear.

Evidence for the impact of self-help and supported therapy on carer depression, anxiety and 'burden' was inconsistent.[36] Similarly, there was inconsistent evidence about the effect of individual psychosocial support interventions on 'carer burden' and depression.[37] Such individual psychosocial support interventions may improve carer stress and economic burden but this evidence was only identified in a single study in one review. There was no evidence of an effect on the outcome 'role strain'. Group psychosocial support interventions may improve carer stress, coping and knowledge but evidence was mixed for the outcomes of depression, 'carer burden' and 'role strain'.[37]

Finally, one review reported evidence about web-based educational interventions. Components of these educational interventions reported in the review included nurse and peer-led support to answer questions by email, provision of information on websites, and training in relaxation and exercise skills. There was no consistent evidence that these interventions were more beneficial than usual care.[36]

Key gaps in evidence about carer interventions

Supplementary materials table S4 maps intervention types against the outcomes reported in the reviews. Evidence for respite and psychosocial interventions was most wide ranging.[36-40] By contrast, there were notable gaps in evidence for cognitive, educational and multicomponent interventions. Outcomes reported less often were economic burden, relationships, and physical health.

Discussion

For peer review only - http://bmjopen.bmj.com/site/about/guidelines.xhtml

Older people's need for unpaid care is predicted to rise with population ageing.[2] Supporting the health and wellbeing of people providing unpaid care for older people, and mitigating the social and financial impacts of caring, should be a public health priority. Our rapid review of systematic reviews mapped current evidence to provide an overview of what is known, as well as identifying key gaps, about the health, social and financial impact of unpaid caring for older people, and how this group of carers can be best supported.

The challenges and demands of unpaid caring for older people are well documented.[9 10 12 13] However, the current evidence fails to fully quantify the consequences of caring for older people on carers' health and wellbeing. Clear measurement of outcomes, with comparison to the general population, is needed to allow the full extent of this impact to be understood.

The evidence reviewed here highlights gaps in our understanding and suggests that a wider consideration of relevant outcomes is needed. Research into the impact of caring and effectiveness of carer interventions must consider outcomes beyond mental health. In particular, physical health was largely neglected in these reviews about caring for older populations. A bias towards mental and psychological, rather than physical, health outcomes has also been noted by others in evidence about the consequences of caring.[41-43] Other reviews about the impact of caring at all ages report that carers are at increased risk of musculoskeletal conditions, cardiovascular disease, generalised cognitive deterioration and poor sleep.[41 44-46] Yet there is a clear gap in these outcomes in systematic reviews that focus on caring for older people. Similarly, we know that unpaid caring can lead to a loss of employment or a reduction in working hours.[47-49 50] Some carers face the demands of managing caring responsibilities alongside employment.[51] There are likely to be related impacts on financial and social wellbeing, but these outcomes were also absent in this evidence. Future research must address the full breadth of potential consequences of being a carer, with consideration of these important but overlooked outcomes.

For peer review only - http://bmjopen.bmj.com/site/about/guidelines.xhtml

Another limitation of the current evidence base is the oft-used outcome ‘carer burden’. This is an ambiguous and contentious term,[52] and input from our stakeholder group confirmed the unhelpful nature of this concept. We recommend that future evaluation and reviews avoid this outcome and focus on more meaningful and specific measures of impact.

A final limitation of the evidence relates to whether chosen outcomes are adequate to capture the likely impact of interventions. Poor choice of outcomes may underestimate or overlook the ways in which an intervention can effectively support and benefit carers of older people. This was most apparent in the reviews of respite interventions. Despite respite being valued by carers, there was no evidence that it improved carers’ mental health or ‘carer burden’. Yet respite alone cannot be expected to improve mental health, with other therapeutic support necessary to address these sorts of needs. There may also be outcomes where appropriate respite, even in the short-term, may show beneficial effects (e.g. stabilising carers’ physical illness or injury). Evaluations could offer a more meaningful understanding of how an intervention can support carers by giving greater consideration to the outcomes measured and the potential pathways to impact.

Overall, our review of reviews indicates that a more robust and comprehensive evidence base is needed to inform policy and practice for supporting carers of older people. One mechanism to achieve this is by conceptualising unpaid caring as a social determinant of health. This is possible with an adaption of Dahlgren and White’s (1991) model (figure 2).[53] Working and living conditions are acknowledged as social determinants of health. Highlighting the similar role of unpaid caring would be helpful here; caring responsibilities and their consequences shape health and are socially patterned. In an area of growing unmet need, framing unpaid caring as a social determinant of health may focus the attention of policymakers and drive the development of evidence-based interventions.

Figure 2 here

Moving forward, we propose the following future research priorities:

For peer review only - http://bmjopen.bmj.com/site/about/guidelines.xhtml

1. A high quality comprehensive systematic review about the impact of caring for older people on carers' physical health and social and financial wellbeing outcomes, supported by a logic model to identify relevant outcomes not captured in existing reviews

2. Clear quantification of the prevalence and severity of mental health outcomes and other illnesses experienced by carers of older people, with comparisons to the general population to highlight the extent of any impact on health and well-being, and potential inequalities between carers and non-carers

3. Robust development and evaluation of promising interventions for carers of older people, with consideration of pathways to impact and a broader range of relevant outcomes

The priorities outlined above have potential to address gaps in the current evidence base identified in our review, and to improve the overall quality and scope of evidence about caring for older people.

Strengths and limitations

Our chosen method was a review of published systematic reviews to avoid duplicating existing work. This is a valuable approach that has highlighted key gaps in the evidence base about caring for older people. A limitation of this method is that primary studies, which have not yet been subjected to a systematic review, are inevitably excluded. However, nine of the 12 included systematic reviews were published within the last three years (2017-2019) and the remainder published between 2007 and 2012. The number of primary studies potentially excluded by using this method is therefore likely to be low.

Conclusion

Current evidence about caring for older people lacks the necessary detail to fully quantify the impact of unpaid caring on carers, and to identify how this population of

For peer review only - http://bmjopen.bmj.com/site/about/guidelines.xhtml

carers should be supported. Classification of unpaid caring as a social determinant of health could be an effective lever to bring greater focus to this population in an area of growing unmet need. Further research is needed to identify suitable interventions, in order to support evidence-based policymaking and practice.

For peer review only

For peer review only - http://bmjopen.bmj.com/site/about/guidelines.xhtml

1. Office for National Statistics. Living longer: how our population is changing and why it matters, 2018.
2. Wittenburg R, Comas-Herrera A, Pickard L, et al. Future demand for long-term care in the UK. London: Joseph Rowntree Foundation, 2004.
3. Association LG. Adult Social Care Funding: 2016 State of the Nation Report: Local Government Association; 2016 [Available from: http://www.local.gov.uk/documents/10180/7632544/1+24+ASCF+state+of+the+nation+2016_WEB.pdf/e5943f2d-4dbd-41a8-b73e-da0c7209ec12 accessed 22/03/2017).
4. Commission CQ. The state of health and social care in England 2017/2018: Care Quality Commission, 2018.
5. Age UK. Age UK General Election Manifesto 2019, 2019.
6. Carers UK. Facts about carers, August 2019 Policy Briefing, 2019.
7. Carers UK. Key facts about carers and the people they care for 2015 [Available from: https://carers.org/key-facts-about-carers-and-people-they-care accessed 25.02.2020.
8. Age UK. Older carers left to fill the gap as our social care system crumbles: Age UK, 2017.
9. Giraldo-Rodríguez L, Guevara-Jaramillo N, Agudelo-Botero M, et al. Qualitative exploration of the experiences of informal care-givers for dependent older adults in Mexico City. *Ageing and Society* 2018;39(11):2377-96. doi: 10.1017/s0144686x18000478
10. Saletti-Cuesta L, Tutton E, Langstaff D, et al. Understanding informal carers' experiences of caring for older people with a hip fracture: a systematic review of qualitative studies. *Disabil Rehabil* 2018;40(7):740-50. doi: 10.1080/09638288.2016.1262467 [published Online First: 2016/12/16]
11. Williams A, Sethi B, Duggleby W, et al. A Canadian qualitative study exploring the diversity of the experience of family caregivers of older adults with multiple chronic conditions using a social location perspective. *Int J Equity Health* 2016;15:40. doi: 10.1186/s12939-016-0328-6 [published Online First: 2016/03/05]
12. McGarry J, Arthur A. Informal caring in late life: a qualitative study of the experiences of older carers. *Journal of Advanced Nursing* 2008;33(2):182-89. doi: 10.1111/j.1365-2648.2001.01651.x
13. Rodger D, Neill MO, Nugent L. Informal carers' experiences of caring for older adults at home: a phenomenological study. *Br J Community Nurs* 2015;20(6):280-5. doi: 10.12968/bjcn.2015.20.6.280 [published Online First: 2015/06/05]
14. Carers UK, Age UK. Caring into later life: Carers UK, Age UK, 2015.
15. Carers UK. In Poor Health: Carers UK, 2004.
16. Al-Janabi H, Coast J, Flynn TN. What do people value when they provide unpaid care for an older person? A meta-ethnography with interview follow-up. *Soc Sci Med* 2008;67(1):111-21. doi: 10.1016/j.socscimed.2008.03.032 [published Online First: 2008/04/26]
17. Yeandle S. Caring for our carers: an international perspective on policy developments in the UK. *Juncture* 2016;23(1):57-62.

For peer review only

For peer review only - http://bmjopen.bmj.com/site/about/guidelines.xhtml

18. National Institute of Health & Care Excellence. Supporting adult carers, NICE Guideline 150 2020 [Available from: https://www.nice.org.uk/guidance/ng150 accessed 25.02.2020.
19. Dickinson C, Dow J, Gibson G, et al. Psychosocial intervention for carers of people with dementia: What components are most effective and when? A systematic review of systematic reviews. *International Psychogeriatrics* 2017;29(1):31-43.
20. Gilhooly KJ, Gilhooly ML, Sullivan MP, et al. A meta-review of stress, coping and interventions in dementia and dementia caregiving. *BMC Geriatr* 2016;16:106. doi: 10.1186/s12877-016-0280-8 [published Online First: 2016/05/20]
21. Huis In Het Veld JG, Verkaik R, Mistiaen P, et al. The effectiveness of interventions in supporting self-management of informal caregivers of people with dementia; a systematic meta review. *BMC Geriatr* 2015;15:147. doi: 10.1186/s12877-015-0145-6 [published Online First: 2015/11/13]
22. Thomas S, Dalton J, Harden M, et al. Updated meta-review of evidence on support for carers. *NIHR Journals Library Health Services and Delivery Research* 2017;3:03.
23. Tricco AC, Antony J, Zarin W, et al. A scoping review of rapid review methods. *BMC Medicine* 2015;13(1):224. doi: 10.1186/s12916-015-0465-6
24. Haby MM, Chapman E, Clark R, et al. What are the best methodologies for rapid reviews of the research evidence for evidence-informed decision making in health policy and practice: a rapid review. *Health Res Policy Syst* 2016;14(1):83. doi: 10.1186/s12961-016-0155-7 [published Online First: 2016/11/26]
25. Canadian Agency for Drugs and Technologies in Health. Strings Attached: CADTH's Database Search Filters 2019 [Available from: https://www.cadth.ca/resources/finding-evidence/strings-attached-cadths-database-search-filters#syst accessed 28.02.2020.
26. Centre for Reviews and Dissemination (UK). Database of Abstracts of Reviews of Effects (DARE): Quality-assessed Reviews York (UK)1995 [Available from: https://www.ncbi.nlm.nih.gov/books/NBK285222/.
27. Ouzzani M, Hammady H, Fedorowicz Z, et al. Rayyan—a web and mobile app for systematic reviews. *Systematic Reviews* 2016;5(1):210. doi: 10.1186/s13643-016-0384-4
28. Whiting P, Savovic J, Higgins JP, et al. ROBIS: A new tool to assess risk of bias in systematic reviews was developed. *J Clin Epidemiol* 2016;69:225-34. doi: 10.1016/j.jclinepi.2015.06.005 [published Online First: 2015/06/21]
29. Amer Nordin A, Mohd Hairi F, Choo WY, et al. Care Recipient Multimorbidity and Health Impacts on Informal Caregivers: A Systematic Review. *Gerontologist* 2019;59(5):e611-e28. doi: 10.1093/geront/gny072 [published Online First: 2018/07/10]
30. Bom J, Bakx P, Schut F, et al. The Impact of Informal Caregiving for Older Adults on the Health of Various Types of Caregivers: A Systematic Review. *The Gerontologist* 2018;59(5):629-42.
31. Ge L, Mordiffi SZ. Factors associated with higher caregiver burden among family caregivers of elderly cancer patients: A systematic review. *Cancer Nursing* 2017;40(6):471-78.

For peer review only - http://bmjopen.bmj.com/site/about/guidelines.xhtml

32. Jansen L, Dauphin S, van den Akker M, et al. Prevalence and predictors of psychosocial problems in informal caregivers of older cancer survivors - A systematic review: Still major gaps in current research. *European Journal of Cancer Care* 2018;27(6):1-13. doi: 10.1111/ecc.12899
33. Namasivayam-MacDonald AM, Shune SE. The Burden of Dysphagia on Family Caregivers of the Elderly: A Systematic Review. *Geriatrics (Basel)* 2018;3(2):10. doi: 10.3390/geriatrics3020030 [published Online First: 2019/04/24]
34. Ringer T, Hazzan AA, Agarwal A, et al. Relationship between family caregiver burden and physical frailty in older adults without dementia: a systematic review. *Syst Rev* 2017;6(1):55. doi: 10.1186/s13643-017-0447-1 [published Online First: 2017/03/16]
35. Domingues NS, Verreault P, Hudon C. Reducing Burden for Caregivers of Older Adults With Mild Cognitive Impairment: A Systematic Review. *Am J Alzheimers Dis Other Demen* 2018;33(7):401-14. doi: 10.1177/1533317518788151 [published Online First: 2018/07/26]
36. Guay C, Auger C, Demers L, et al. Components and Outcomes of Internet-Based Interventions for Caregivers of Older Adults: Systematic Review. *J Med Internet Res* 2017;19(9):e313. doi: 10.2196/jmir.7896 [published Online First: 2017/09/21]
37. Lopez-Hartmann M, Wens J, Verhoeven V, et al. The effect of caregiver support interventions for informal caregivers of community-dwelling frail elderly: a systematic review. *Int J Integr Care* 2012;12:e133. doi: 10.5334/ijic.845 [published Online First: 2013/04/18]
38. Mason A, Weatherly H, Spilsbury K, et al. The effectiveness and cost-effectiveness of respite for caregivers of frail older people. *J Am Geriatr Soc* 2007;55(2):290-9. doi: 10.1111/j.1532-5415.2006.01037.x [published Online First: 2007/02/17]
39. Murfield J, Moyle W, Jones C, et al. Self-Compassion, Health Outcomes, and Family Carers of Older Adults: An Integrative Review. *Clin Gerontol* 2019:1-14. doi: 10.1080/07317115.2018.1560383 [published Online First: 2019/01/08]
40. Shaw C, McNamara R, Abrams K, et al. Systematic review of respite care in the frail elderly. *Health Technol Assess* 2009;13(20):1-224, iii. doi: 10.3310/hta13200 [published Online First: 2009/04/28]
41. Cottagiri S, Sykes P. Key health impacts and support systems for informal carers in the UK: A thematic review *Journal of Health and Social Sciences* 2019 doi: doi10.19204/2019/kyhl11
42. Pinquart M, Sörensen S. Correlates of Physical Health of Informal Caregivers: A Meta-Analysis. *The Journals of Gerontology: Series B* 2007;62(2):P126-P37. doi: 10.1093/geronb/62.2.P126
43. Clancy RL, Fisher GG, Daigle KL, et al. Eldercare and Work Among Informal Caregivers: A Multidisciplinary Review and Recommendations for Future Research. *Journal of Business and Psychology* 2020;35(1):9-27. doi: 10.1007/s10869-018-9612-3
44. Fonareva I, Oken BS. Physiological and functional consequences of caregiving for relatives with dementia. *Int Psychogeriatr* 2014;26(5):725-47. doi: 10.1017/s1041610214000039 [published Online First: 2014/02/11]

For peer review only - http://bmjopen.bmj.com/site/about/guidelines.xhtml

45. Romero-Martínez Á, Hidalgo-Moreno G, Moya-Albiol L. Neuropsychological consequences of chronic stress: the case of informal caregivers. *Aging & Mental Health* 2020;24(2):259-71. doi: 10.1080/13607863.2018.1537360
46. Vitaliano PP, Zhang J, Scanlan JM. Is caregiving hazardous to one's physical health? A meta-analysis. *Psychological bulletin* 2003;129(6):946-72. doi: 10.1037/0033-2909.129.6.946 [published Online First: 2003/11/06]
47. Carers UK. In sickness and in health, 2012.
48. King D, Pickard L. When is a carer's employment at risk? Longitudinal analysis of unpaid care and employment in midlife in England. *Health Soc Care Community* 2013;21(3):303-14. doi: 10.1111/hsc.12018 [published Online First: 2013/01/30]
49. Van Houtven CH, Coe NB, Skira MM. The effect of informal care on work and wages. *Journal of Health Economics* 2013;32(1):240-52. doi: https://doi.org/10.1016/j.jhealeco.2012.10.006
50. Carers UK. Juggling work and unpaid care, 2019.
51. Vlachantoni A. The demographic characteristics and economic activity patterns of carers over 50: Evidence from the English Longitudinal Study of Ageing. *Population trends* 2010;141(141):51-73.
52. Bastawrous M. Caregiver burden--a critical discussion. *Int J Nurs Stud* 2013;50(3):431-41. doi: 10.1016/j.ijnurstu.2012.10.005 [published Online First: 2012/11/08]
53. Dahlgren G, Whitehead M. European strategies for tackling social inequities in health: Levelling up Part 2. *WHO Collaborating Centre for Policy Research on Social Determinants of Health, University of Liverpool* 1991

For peer review only - http://bmjopen.bmj.com/site/about/guidelines.xhtml

Figure 1.PRISMA flowchart of study selection

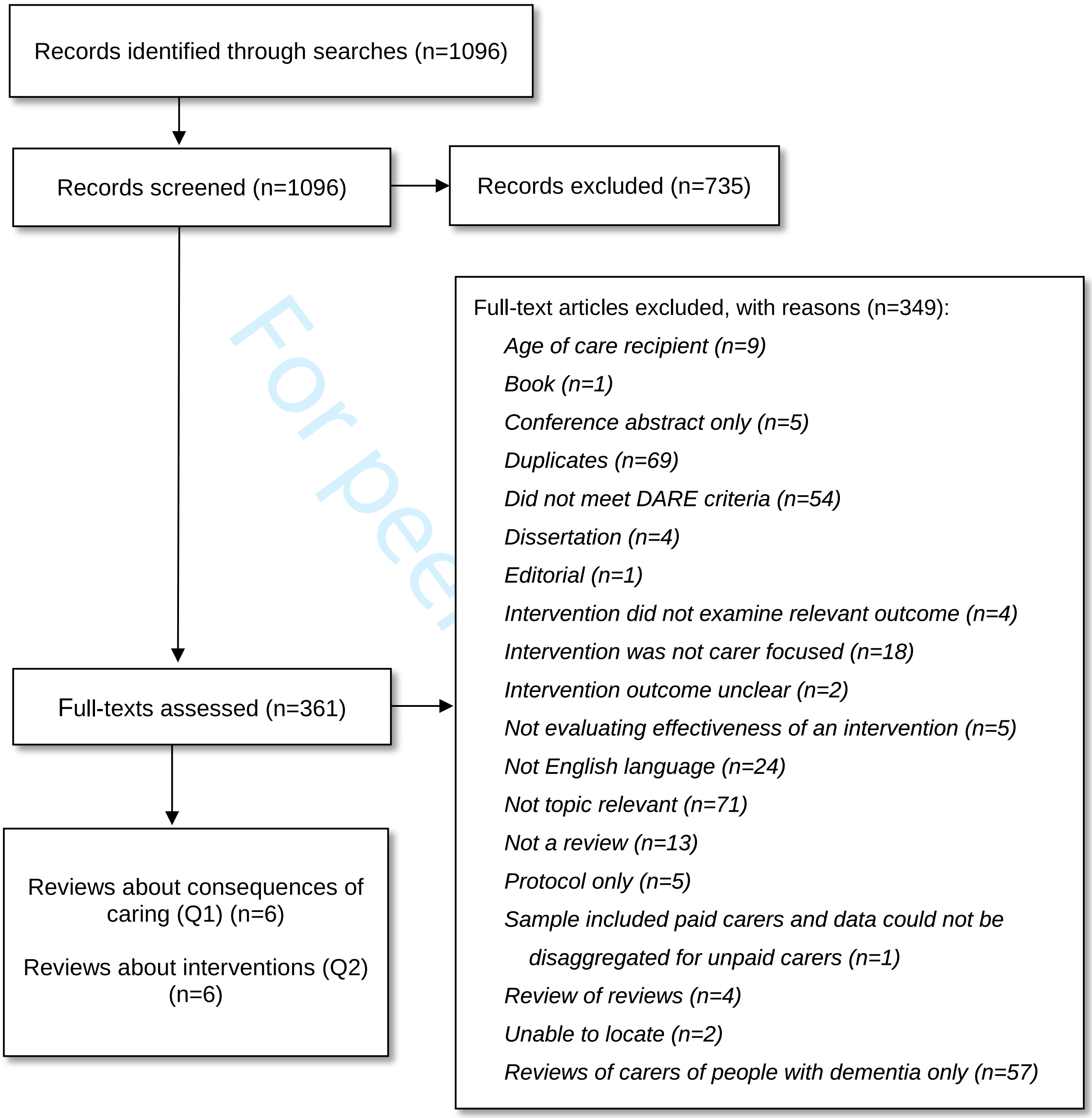

For peer review only - http://bmjopen.bmj.com/site/about/guidelines.xhtml

Figure 2. Unpaid caring as a social determinant of health: an adaptation of Dalgren & Whitehead's (1991) model

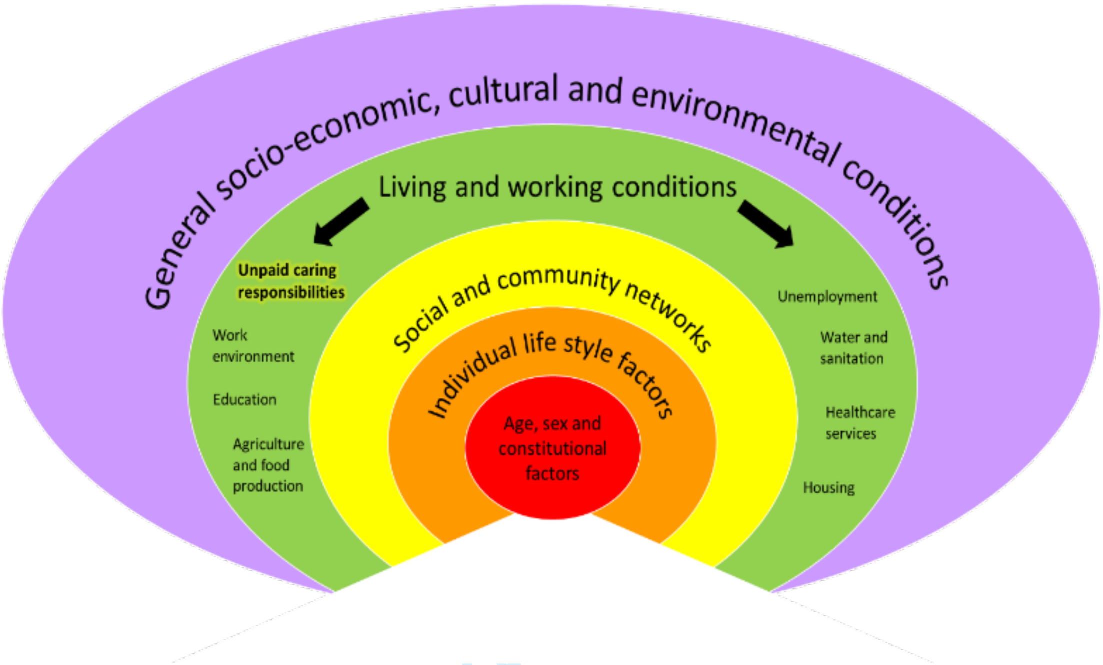

For peer review only - http://bmjopen.bmj.com/site/about/guidelines.xhtml

For peer review only - http://bmjopen.bmj.com/site/about/guidelines.xhtml

Supplementary materials

Appendix A: Search strategy as applied to MEDLINE

Database(s): **Ovid MEDLINE(R)** 1946 to January Week 2 2020; Searched 21st January

#	Searches	Results
1	meta-analysis.pt.	109635
2	meta-analysis/ or systematic review/ or meta-analysis as topic/ or "meta analysis (topic)"/ or "systematic review (topic)"/ or exp technology assessment, biomedical/	205585
3	((systematic* adj3 (review* or overview* or analys*)) or (methodologic* adj3 (review* or overview* or analys*))).ti,ab,kf,kw.	150031
4	(((quantitative or narrative) adj3 (review* or overview* or synthes*)) or (research adj3 (integrati* or overview*))).ti,ab,kf,kw.	17348
5	((integrative adj3 (review* or overview*)) or (collaborative adj3 (review* or overview*)) or (pool* adj3 analy*)).ti,ab,kf,kw.	20727
6	(data synthes* or data extraction* or data abstraction*).ti,ab,kf,kw.	20899
7	(handsearch* or hand search*).ti,ab,kf,kw.	7850
8	(mantel haenszel or peto or der simonian or dersimonian or fixed effect* or latin square*).ti,ab,kf,kw.	21652
9	(met analy* or metanaly* or technology assessment* or HTA or HTAs or technology overview* or technology appraisal*).ti,ab,kf,kw.	7271
10	(meta regression* or metaregression*).ti,ab,kf,kw.	6630
11	(meta-analy* or metaanaly* or systematic review* or biomedical technology assessment* or bio-medical technology assessment*).mp,hw.	235567
12	(medline or cochrane or pubmed or medlars or embase or cinahl).ti,ab,hw.	166667
13	(cochrane or (health adj2 technology assessment) or evidence report).jw.	18482
14	(comparative adj3 (efficacy or effectiveness)).ti,ab,kf,kw.	10523
15	(outcomes research or relative effectiveness).ti,ab,kf,kw.	7615
16	((indirect or indirect treatment or mixed-treatment) adj comparison*).ti,ab,kf,kw.	1649
17	((meta-narrative or meta-ethnograph* or mixed method* or critical or thematic or realist or framework) adj3 (review* or synthes*)).ti,ab,kf,kw.	20949
18	or/1-17	395639
19	((carer* or caregiver* or caring or care partner* or "family care" or "spousal care" or "unpaid care" or "informal care") adj5 (older or senior* or elder* or dement* or alzheimer*)).ti,ab.	9395
20	18 and 19	613

For peer review only

Table S1. Abbreviated risk of bias criteria, adapted from the ROBIS tool[1]

1. Can searches be considered exhaustive and thus prone to minimal bias?
2. Are review criteria clear, and thus prone to minimal selection bias?
3. Is the quality assessment incorporated into the synthesis to prioritise more robust evidence, or considered in relation to the conclusions drawn?
4. Is the synthesis transparent and clear: sufficient study details are reported; synthesised evidence is referenced appropriately?
5. Do review findings and synthesis avoid emphasising results based only on statistical significance?

For peer review only

Table S2. The consequences of caring for older populations: summary of evidence by review risk of bias

Study	Type of consequence/impact reported					Evidence about which groups may be at greater risk
	**Health (physical and mental)**	**Social**	**Finance**	**Quality of life**	**Other**	
	Evidence from reviews with a moderate risk of bias					
Ge [31]	'BURDEN' (PREVALENCE)<br><br>Across 3 studies, the proportion of carers reporting levels of 'carer burden' varied from 37% to 100%.<br><br>'BURDEN' (SEVERITY)<br><br>The percentage of carers who reported high 'burden' ranged from 1% to greater than 35% (across 4 studies).	NA	NA	NA	NA	SEX<br><br>Limited evidence of an association between sex of carer (male) and higher 'carer burden'.<br><br>AGE<br><br>Younger carer age was associated with higher 'carer burden'.<br><br>SES<br><br>Limited evidence that lack of social support and insufficient financial support were factors contributing to higher 'carer burden'.
Ringer[34]	'BURDEN', DEPRESSION, ANXIETY<br><br>Carers of frail older adults experienced 'burden', depression and anxiety, but	NA	NA	NA	NA	None

For peer review only

	it was not possible to quantify this from included studies. Evidence from one included study indicated that the 'burden' experienced by carers of frail older people was less than that of carers of those with cancer, diabetes and dementia.					
	Evidence from reviews with a high risk of bias					
Amer [29]	DEPRESSION (SEVERITY)<br><br>Evidence about the level of depression experienced by carers differed across studies; levels were judged to be both mild to moderate, and severe, although it is not clear how these were defined.<br><br>'BURDEN' (SEVERITY)<br><br>'Carer burden' was judged to be low to moderate, although it is not clear how these are defined.	NA	NA	Carers' quality of life was lower compared to national levels.	Limited findings suggest an association between carers' perceived difficulty in assisting care recipients and differences in cultural values.	None
Bom [30]	DEPRESSION (PREVALENCE)<br><br>Caring was associated with higher prevalence of depressive feelings and lower mental health scores.<br><br>PHYSICAL HEALTH	NA	NA	NA	NA	SEX<br><br>Impact of caring on health is greater for females and those married.

	There was mixed evidence about impact on physical health; review authors suggest this reflects different outcome measures.				
Jansen [32]	DISTRESS AND ANXIETY (SEVERITY)<br><br>There was limited evidence showing greater distress and anxiety in carers of older cancer survivors compared to the general population.<br><br>SELF-ESTEEM<br><br>There was limited evidence that caring was associated with a high self-esteem.<br><br>DEPRESSION<br><br>Evidence of impact on depression was reported to be varied (although full details are not reported). Authors suggest that this was due to the variety of instruments used to measure the outcome.<br><br>'BURDEN' (PREVALENCE)		Limited evidence shows an association between lower quality of life and carers of older cancer survivors compared with the general population	There was limited evidence that carers reported difficulties in talking to the care recipients about their illness, suggesting communicatio n issues	SEX<br><br>One study reported that being a female carer was a predictor for 'burden' while another study found that being a male spousal carer was a risk factor for 'burden'.

For peer review only

	Limited evidence indicated 40% of carers experienced high 'carer burden'.
	STRESS (SEVERITY)
	Limited evidence that carers experience higher levels of stress than the UK general adult population

For peer review only

Namasivayam [33]	'BURDEN' (SEVERITY)<br><br>Dysphagia in older care recipients is associated with increased 'carer burden'.	NA	NA	NA	NA

For peer review only

Table **S3. Interventions for carers: summary of evidence by review risk of bias**

Study	Interventions for carers of older people	
	**Findings**	**Sub-group analysis**
	Evidence from reviews with a low risk of bias	
Shaw [40]	Pooled analyses indicated respite care had no effect on 'carer burden' and anxiety, and little effect on carer depression. Some evidence indicated that respite was associated with worse carer quality of life, although the review authors note there were potential sources of bias in these studies. Other evidence indicated respite may improve the carer-recipient relationship. Home help-based respite may improve carers' morale.	None reported.
Mason [38]	There was no consistent evidence to indicate respite care was beneficial to carers. Pooled analysis indicated no effect on 'carer burden', and a small improvement in depression, although authors note a methodological flaw which undermines the reliability of this finding. However, satisfaction levels were generally high for all types of respite compared to usual care.	*Although the authors report findings from one study about outcomes for sub-groups, it is not clear if this refers to the carer or recipient. Other sub-group findings reported relate to likelihood of the carer 'institutionalising' the care recipient.*
	Evidence from reviews with a moderate risk of bias	
Murfield [39]	There was limited evidence (1 study) that a mindfulness stress reduction intervention may improve carer depression and anxiety but not other outcomes. There was limited evidence (1 study) that a yoga + meditation based intervention improved carer self-compassion and quality of life.	None reported.
	Evidence from reviews with a high risk of bias	
Domingues [35a]	There was limited and mixed evidence for a cognitive intervention and a multicomponent intervention.	None reported.
Guay [36]	There was no consistent evidence to indicate web-based education interventions were beneficial to carers, and mixed evidence for therapy interventions.	None reported.
Lopez-Hartman [37]	There was inconsistent evidence about whether individual and group psychosocial support interventions are beneficial for carers, with improvements to some outcomes but not others.	One study reported that carers sharing a household with care recipients may benefit more from an individual psychosocial support group compared to those living apart.

[a]*Other interventions were reported in this review but did not target only the carer and were primarily oriented towards supporting the care recipient.*

For peer review only

Table S4. Reviews reporting outcomes by intervention classification

	INTERVENTION TYPE				
**OUTCOME**	**Respite**	**Psychosocial**	**Cognitive**	**Education**	**Multicomponent**
**'Burden'**	Shaw 2009 Mason 2007	Lopez-Hartman 2012 Guay 2017	Domingues 2018	Guay 2017	Domingues 2018
**Depression/mood**	Shaw 2009	Lopez-Hartman 2012 Guay 2017	Domingues 2018	Guay 2017	Domingues 2018
**Anxiety**	Shaw 2009	Lopez-Hartman 2012 Guay 2017	-	-	-
**Stress, wellbeing, quality of life, 'role strain', morale, self-compassion**	Shaw 2009	Lopez-Hartman 2012 Murfield 2019	-	Guay 2017	
**Health, physical burden, 'other' health**	-	Guay 2017	-	-	-
**Anger, hostility**	Shaw 2009	-	-	-	-
**Knowledge**	-	Lopez-Hartman 2012 Guay 2017	-	-	-
**Coping**	-	Lopez-Hartman 2012 Guay 2017	-	-	-
**Relationships**	Shaw 2009	-	-	-	-
**Satisfaction**	Mason 2007	-	-	-	-
**Economic burden**	-	Lopez-Hartman 2012	-	-	-

For peer review only

PRISMA 2009 Checklist

Section/topic	#	Checklist item	Reported on page #
**TITLE**			
Title	1	Identify the report as a systematic review, meta-analysis, or both.	1
**ABSTRACT**			
Structured summary	2	Provide a structured summary including, as applicable: background; objectives; data sources; study eligibility criteria, participants, and interventions; study appraisal and synthesis methods; results; limitations; conclusions and implications of key findings; systematic review registration number.	3
**INTRODUCTION**			
Rationale	3	Describe the rationale for the review in the context of what is already known.	5-6
Objectives	4	Provide an explicit statement of questions being addressed with reference to participants, interventions, comparisons, outcomes, and study design (PICOS).	6
**METHODS**			
Protocol and registration	5	Indicate if a review protocol exists, if and where it can be accessed (e.g., Web address), and, if available, provide registration information including registration number.	NA
Eligibility criteria	6	Specify study characteristics (e.g., PICOS, length of follow-up) and report characteristics (e.g., years considered, language, publication status) used as criteria for eligibility, giving rationale.	7-8, Table 1
Information sources	7	Describe all information sources (e.g., databases with dates of coverage, contact with study authors to identify additional studies) in the search and date last searched.	8
Search	8	Present full electronic search strategy for at least one database, including any limits used, such that it could be repeated.	Supplementary materials
Study selection	9	State the process for selecting studies (i.e., screening, eligibility, included in systematic review, and, if applicable, included in the meta-analysis).	8
Data collection process	10	Describe method of data extraction from reports (e.g., piloted forms, independently, in duplicate) and any processes for obtaining and confirming data from investigators.	8-9
Data items	11	List and define all variables for which data were sought (e.g., PICOS, funding sources) and any assumptions and simplifications made.	8-9
Risk of bias in individual studies	12	Describe methods used for assessing risk of bias of individual studies (including specification of whether this was done at the study or outcome level), and how this information is to be used in any data synthesis.	8-9
Summary measures	13	State the principal summary measures (e.g., risk ratio, difference in means).	NA
Synthesis of results	14	Describe the methods of handling data and combining results of studies, if done, including measures of consistency (e.g., I^2) for each meta-analysis.	9

For peer review only

For peer review only - http://bmjopen.bmj.com/site/about/guidelines.xhtml

PRISMA 2009 Checklist

Section/topic	#	Checklist item	Reported on page #
Risk of bias across studies	15	Specify any assessment of risk of bias that may affect the cumulative evidence (e.g., publication bias, selective reporting within studies).	NA
Additional analyses	16	Describe methods of additional analyses (e.g., sensitivity or subgroup analyses, meta-regression), if done, indicating which were pre-specified.	NA
**RESULTS**			
Study selection	17	Give numbers of studies screened, assessed for eligibility, and included in the review, with reasons for exclusions at each stage, ideally with a flow diagram.	9, and figure 1
Study characteristics	18	For each study, present characteristics for which data were extracted (e.g., study size, PICOS, follow-up period) and provide the citations.	9, tables 2 and 3
Risk of bias within studies	19	Present data on risk of bias of each study and, if available, any outcome level assessment (see item 12).	10-11, supplementary materials table S2 and S3
Results of individual studies	20	For all outcomes considered (benefits or harms), present, for each study: (a) simple summary data for each intervention group (b) effect estimates and confidence intervals, ideally with a forest plot.	Supplementary materials tables S2 and S3
Synthesis of results	21	Present results of each meta-analysis done, including confidence intervals and measures of consistency.	NA – no meta analysis
Risk of bias across studies	22	Present results of any assessment of risk of bias across studies (see Item 15).	10-11, supplementary materials table S2 and S3
Additional analysis	23	Give results of additional analyses, if done (e.g., sensitivity or subgroup analyses, meta-regression [see Item 16]).	NA
**DISCUSSION**			
Summary of evidence	24	Summarize the main findings including the strength of evidence for each main outcome; consider their relevance to key groups (e.g., healthcare providers, users, and policy makers).	12-15
Limitations	25	Discuss limitations at study and outcome level (e.g., risk of bias), and at review-level (e.g., incomplete retrieval of identified research, reporting bias).	15

For peer review only

For peer review only - http://bmjopen.bmj.com/site/about/guidelines.xhtml

PRISMA 2009 Checklist

Conclusions	26	Provide a general interpretation of the results in the context of other evidence, and implications for future research.	12-15
**FUNDING**			
Funding	27	Describe sources of funding for the systematic review and other support (e.g., supply of data); role of funders for the systematic review.	Title page

From: Moher D, Liberati A, Tetzlaff J, Altman DG, The PRISMA Group (2009). Preferred Reporting Items for Systematic Reviews and Meta-Analyses: The PRISMA Statement. PLoS Med 6(7): e1000097. doi:10.1371/journal.pmed1000097

For more information, visit: **www.prisma-statement.org**.

For peer review only

For peer review only - http://bmjopen.bmj.com/site/about/guidelines.xhtml

What are the consequences of caring for older people and what interventions are effective for supporting unpaid carers? A rapid review of systematic reviews

Journal:	*BMJ Open*
Manuscript ID	bmjopen-2020-046187.R1
Article Type:	Original research
Date Submitted by the Author:	09-Aug-2021
Complete List of Authors:	Spiers, Gemma; Newcastle University, Population & Health Sciences Institute<br>Liddle, Jennifer; Newcastle University, Population & Health Sciences Institute; Newcastle University, Population & Health Sciences Institute<br>Kunonga, Tafadzwa; Newcastle University, Population & Health Sciences Institute<br>Whitehead, Orla; Newcastle University, Population Health Sciences Institute<br>Beyer, Fiona ; Newcastle University, Population & Health Sciences Institute<br>Stow, Daniel; University of Newcastle upon Tyne, Population and Health Sciences Institute; Newcastle University<br>Welsh, Claire; Newcastle University, Population & Health Sciences Institute<br>Ramsay, Sheena; Newcastle University, Population & Health Sciences Institute<br>Craig, Dawn; Newcastle University, Health Economics Group, Population & Health Sciences Institute<br>Hanratty, Barbara; Newcastle University, Population & Health Sciences Institute
**Primary Subject Heading**:	Health policy
Secondary Subject Heading:	Geriatric medicine, Health services research
Keywords:	Health policy < HEALTH SERVICES ADMINISTRATION & MANAGEMENT, GERIATRIC MEDICINE, PRIMARY CARE

SCHOLARONE™ Manuscripts

I, the Submitting Author has the right to grant and does grant on behalf of all authors of the Work (as defined in the below author licence), an exclusive licence and/or a non-exclusive licence for contributions from authors who are: i) UK Crown employees; ii) where BMJ has agreed a CC-BY licence shall apply, and/or iii) in accordance with the terms applicable for US Federal Government officers or employees acting as part of their official duties; on a worldwide, perpetual, irrevocable, royalty-free basis to BMJ Publishing Group Ltd ("BMJ") its licensees and where the relevant Journal is co-owned by BMJ to the co-owners of the Journal, to publish the Work in this journal and any other BMJ products and to exploit all rights, as set out in our licence.

The Submitting Author accepts and understands that any supply made under these terms is made by BMJ to the Submitting Author unless you are acting as an employee on behalf of your employer or a postgraduate student of an affiliated institution which is paying any applicable article publishing charge ("APC") for Open Access articles. Where the Submitting Author wishes to make the Work available on an Open Access basis (and intends to pay the relevant APC), the terms of reuse of such Open Access shall be governed by a Creative Commons licence – details of these licences and which Creative Commons licence will apply to this Work are set out in our licence referred to above.

Other than as permitted in any relevant BMJ Author's Self Archiving Policies, I confirm this Work has not been accepted for publication elsewhere, is not being considered for publication elsewhere and does not duplicate material already published. I confirm all authors consent to publication of this Work and authorise the granting of this licence.

For peer review only

What are the consequences of caring for older people and what interventions are effective for supporting unpaid carers? A rapid review of systematic reviews

Spiers, Gemma [a] * gemma-frances.spiers@newcastle.ac.uk

Liddle, Jennifer [a, b]

Kunonga, Tafadzwa Patience [a]

Whitehead, Ishbel Orla [a]

Beyer, Fiona [c]

Stow, Daniel [a]

Welsh, Claire [a]

Ramsay, Sheena E. [c]

Craig, Dawn [c]

Hanratty, Barbara [a]

[a] Population and Health Sciences Institute, Newcastle University, Campus for Ageing & Vitality, Newcastle upon Tyne, NE4 5PL
[b] Centre for Digital Citizens, Newcastle University, Newcastle Helix, Newcastle upon Tyne, NE4 5TG
[c] Population and Health Sciences Institute, Newcastle University, Baddiley-Clark, Newcastle upon Tyne, NE2 4AX

*Corresponding author

Word count: 3485
References: 53
Tables: 3 (plus four in supplementary materials)
Figures: 2

For peer review only

For peer review only - http://bmjopen.bmj.com/site/about/guidelines.xhtml

What are the consequences of caring for older people and what interventions are effective for supporting unpaid carers? A rapid review of systematic reviews

Objectives: To identify and map evidence about the consequences of unpaid caring for all carers of older people, and effective interventions to support this carer population.

Design: A rapid review of systematic reviews, focused on the consequences for carers of unpaid caring for older people, and interventions to support this heterogeneous group of carers. Reviews of carers of all ages were eligible, with any outcome measures relating to carers' health, social and financial wellbeing. Searches were conducted in MEDLINE, PsycInfo and Epistemonikos (January 2000 - January 2020). Records were screened, and included systematic reviews were quality appraised. Summary data were extracted and a narrative synthesis produced.

Results: Twelve systematic reviews reporting evidence about the consequences of caring for carers (n=6) and assessing the effectiveness of carer interventions (n=6) were included. The review evidence typically focused on mental health outcomes, with little information identified about carers' physical, social and financial wellbeing. Clear estimates of the prevalence and severity of carer outcomes, and how these differ between carers and non-carers, were absent. A range of interventions were identified, but there was no strong evidence of effectiveness. In some studies, the choice of outcome measure may underestimate the full impact an intervention.

Conclusions:

Current evidence fails to fully quantify the impacts that caring for older people has on carers' health and wellbeing. Information on social patterning of the consequences of caring is absent. Systematic measurement of a broad range of outcomes, with comparison to the general population, is needed to better understand the true consequences of caring. Classification of unpaid caring as a social determinant of health could be an effective lever to bring greater focus and support to this population. Further work is needed to develop and identify suitable interventions, in order to support evidence-based policymaking and practice.

For peer review only - http://bmjopen.bmj.com/site/about/guidelines.xhtml

Strengths and limitations of this study

- A rapid review of published systematic reviews offers a useful and efficient approach to summarising evidence about caring for older people, without duplicating existing work.
- This approach allowed us to identify key gaps in this evidence base and make clear recommendations for future research.
- A limitation of this method is the exclusion of primary research studies that have not yet been subject to a systematic review.
- Most of the included systematic reviews were published within the last three years, signalling a contemporary evidence base.

For peer review only

For peer review only - http://bmjopen.bmj.com/site/about/guidelines.xhtml

Background

Populations are ageing worldwide. Life expectancy is increasing in many high-income countries, whilst the proportion of those aged 65 and over is projected to increase 16% by 2050.[1] However, longer lives will not necessarily be spent in good health, and population ageing will likely result in an increase in care needs. Indeed, current projections suggest that the number of dependent older people in the UK will increase by 113% by 2051.[2] Current state provision of social care has failed to keep pace with current levels of demand.[3 4] For example, around 1.5 million older people are estimated to have an unmet need for care.[5] The consequences of this gap between care needs and care provision are also likely to be experienced by families and friends who provide unpaid care. Indeed, there are approximately 6.5 million people in the UK providing unpaid care to ill and disabled family members, friends or partners,[6] with an estimated economic contribution of up to £132 billion per year.[7] In this paper, we use the term 'carer' to refer to people providing unpaid care, although we recognise that not every carer identifies themselves as such.[8]

Carers make a critical contribution to health and social care.[9] Yet caring can have profound impacts on the lives of carers. The demands of caring bring emotional, health and financial challenges,[10 11] alongside changes to home and daily routines, work and career, family and social networks.[12-14] Carers are at greater risk of premature death and higher disease prevalence, whilst neglect of their own health care needs is common.[15] Such poor health is likely to be exacerbated by the social isolation, poor information and support, and financial stress that carers experience.[16] Supporting the mental and physical health and wellbeing of carers is essential. It is also critical that any form of support must account for the complexity of caring. For example, six key attributes of the caring experience (for those caring for an older person) have been identified as: getting on with the care recipient; assistance from organisations and the government; support from family and friends; activities outside caring; control; and fulfilment.[17] These attributes reflect the need to consider a broad range of outcomes when designing, implementing and evaluating carer support interventions.

For peer review only - http://bmjopen.bmj.com/site/about/guidelines.xhtml

Recognition of the role and contribution of carers has grown since the 1960s.[18] In the UK, National Institute for Health and Care Excellence (NICE) guidelines for supporting carers were published in 2020, highlighting this population as a policy priority.[19] Evidence informed practice will ensure that the most effective interventions are used to support carers, whilst targeting the most relevant outcomes. As the number of older people requiring care rises, it is important that evidence is relevant to the contribution and support needs of people caring for older populations. Recent umbrella reviews have focused on evidence on interventions for carers more broadly or carers of people with dementia.[20-23] Whilst this is important, there is now a clear need to map the evidence about carers of older people, with a focus on both the consequences of caring *and* the most effective forms of carer support. This will enhance our understanding of how best to support carers of older people.

We undertook a review of systematic reviews to map current evidence about caring for an older person, addressing two key questions:

1. What are the consequences of caring for older people for the health, social and financial wellbeing of carers, and what do we know about how these consequences vary by age, sex, socioeconomic status and geographical location?

2. Which interventions are effective (including consideration of costs) to promote health and wellbeing and access to services amongst these carers?

The aim of this work was to provide an overview of what is known, as well as identifying key gaps, to support evidence informed practice for those caring for older people.

Methods

The approach to this evidence synthesis was a rapid review of published systematic reviews (from here on referred to as 'rapid review'). Preliminary scoping of the literature identified multiple systematic reviews on both the consequences of caring and associated interventions to support those caring for older people. Thus, the

For peer review only - http://bmjopen.bmj.com/site/about/guidelines.xhtml

review of reviews ('umbrella review') approach was most appropriate as an efficient approach to assessing the evidence, without duplicating existing research. Rapid review methodology was employed, which uses a streamlined approach to study selection and synthesis in order to produce a timely overview of evidence.[24 25] The following methods are reported in accordance with Preferred Reporting Items for Systematic Reviews and Meta-Analyses (PRISMA) guidelines.

Search strategy

A search strategy was developed, piloted and refined, combining terms for 'carers' with validated systematic review filters (see supplementary materials).[26] Searches were carried out in MEDLINE, PsycInfo and Epistemonikos (https://www.epistemonikos.org/) in January 2020, and were limited to English language publications published after 2000.

Review criteria

Table 1 outlines the review criteria. Eligible systematic reviews were those that: reported evidence about the consequences of caring, or the effectiveness of interventions for promoting carer health and wellbeing and access to services, where the care recipients were older people. Initial scoping indicated that many reviews *did not* specify an age threshold (e.g. >65 years) for older care recipients. Therefore, no age criteria for care recipients were specified in this rapid review. Instead, reviews were included if the care recipient population was described as older people, or where the recipient population was likely to include older populations. In consultation with a study steering group, reviews that focused solely on carers of people with dementia were excluded, as evidence about dementia caregivers has been summarized in three recently published reviews.[20-22] This exclusion criterion avoided duplication of this recent work and ensured we addressed the research questions for carers of older populations more broadly. All carer populations were eligible with no limits on age and sex.

For peer review only - http://bmjopen.bmj.com/site/about/guidelines.xhtml

As the focus of the (second) review question was about interventions to promote *carers'* health, wellbeing and access to services, reviews of *joint carer and recipient* interventions were excluded. Eligible outcomes were any that related to carers' health, social and financial wellbeing, and access to services.

Reviews published after 2000 in English were included, and reviews of studies with any study design were eligible. Reviews must have met at least three of the five Database of Abstracts of Reviews of Effects (DARE) criteria to be considered a systematic review, with criterion 3 being mandatory.[27] Four out of five DARE criteria is the usual threshold to be considered a systematic review. However, this was lowered to three criteria here, to maximise the number of reviews captured and thus evidence scoped. Criterion 3 was mandatory, as quality assessment of included studies is a key component of identifying the most robust evidence.

For peer review only

For peer review only - http://bmjopen.bmj.com/site/about/guidelines.xhtml

Table 1. Review criteria

	INCLUSION CRITERIA	
	**SYNTHESIS 1: CONSEQUENCES OF CARING**	**SYNTHESIS 2: INTERVENTIONS FOR CARERS**
**POPULATION**	Carers (e.g. unpaid, family, 'informal') of older adults. No age criteria for care recipients is specified, but must be, or likely to include, older populations (e.g. people with dementia).<br><br>Carers include people of all ages, male and female.<br><br>Care recipients (i.e. older adults) include male and female, with any medical diagnosis, impairment, disability, or frailty, and no limits to ethnicity.<br><br>Setting – Care provided in the community, hospital, care home.	
**INTERVENTION**	*Not applicable*	Any carer intervention that is targeted only on the carer (i.e. not a joint carer and care recipient intervention) and which aims to improve carers' health, wellbeing and/or access to services.
**COMPARATOR**	No comparator, or non-carers	Any or no comparator, including usual care
**OUTCOME**	Health status, quality of life, well-being, incident ill-health, admission to hospital, financial wellbeing, poverty, measured changes in material circumstances, social relationships including loneliness, isolation, social support, social networks.	
**STUDY DESIGN**	Systematic reviews (those that meet 3 of 5 DARE criteria). Publication dates 2000-2019. If more recently published systematic reviews include evidence that is also in earlier reviews, the most recent reviews will be prioritised to avoid duplication.<br><br>English language publications	

For peer review only - http://bmjopen.bmj.com/site/about/guidelines.xhtml

Study selection

Records were managed in Rayyan software (https://rayyan.qcri.org), an online platform to manage and assist screening for systematic reviews.[28] Titles and abstracts were screened for relevance: 20% by two researchers independently, and the remaining 80% by a single researcher, as per rapid review methods.[24] For the 20% of records screened jointly, disagreements were resolved by taking forward any records, for which decisions differed, to the next stage of screening. Records that were deemed relevant based on title and abstract were retrieved for full text screening against the review criteria by one researcher.

Data extraction

Separate data extraction forms were developed and piloted for each review question, using an Excel spreadsheet. Review details and characteristics were extracted, including: author, year of publication, number of studies included in the review, carer and care recipient population, type of consequence reported, interventions evaluated, outcomes, and type of synthesis. Review findings and conclusions were summarised, including any sub-group analyses where reported.

Quality appraisal

A full risk of bias assessment is not typically part of a rapid review.[24 25] However, an *indication* of bias was deemed important for this synthesis in order to identify the most robust findings, and obtain an overview of the quality of the evidence base. Thus, an abbreviated risk of bias appraisal was completed for included reviews using an amended version of the Risk of Bias in Systematic Reviews (ROBIS) tool.[29]

This appraisal used five items relating to review methodology that indicate likely risk of bias (supplementary materials table S1). Each item was scored 1 for 'yes' and the total score was summed across the five items. A score of 5 indicates a low risk of bias; 4 indicates a moderate risk of bias; three or less indicates a high risk of bias.

For peer review only - http://bmjopen.bmj.com/site/about/guidelines.xhtml

Data synthesis

A narrative synthesis was used to address each question, with reviews grouped into those reporting caring consequences (question 1) and those reporting effectiveness of carer interventions (question 2). Summary tables were produced to describe review characteristics, the type of evidence identified, and indicative risk of bias. Findings were synthesised: first by the judged risk of bias (i.e. low, moderate and high risk of bias) to prioritise the most robust evidence; and then by the type of consequence (question 1) and type of intervention (question 2). For review question 2, all identified interventions from included reviews were mapped against the outcomes reported to identify key gaps in evidence.

Patient and public involvement

A steering group representing diverse perspectives and organisations provided advice on the content of the review and dissemination, in regular meetings. In particular, we excluded carers of people with dementia from this study after discussion with them.

Findings

Twelve systematic reviews met the review criteria (Figure 1). Six reviews reported evidence about the consequences of caring (Table 2)[30-35] and six reported evidence about carer interventions (Table 3).[36-41] In this section, we present the synthesis of evidence about the consequences of caring for older people (question 1) and the effectiveness of carer interventions (question 2). Evidence is reported according to judged risk of bias to prioritise the most robust findings.

For peer review only - http://bmjopen.bmj.com/site/about/guidelines.xhtml

Table 2. Overview of systematic reviews reporting evidence about the consequences of caring

Study	Date	Number of studies in review	Data range of included studies	Population (care recipient)	Type of consequence/impact reported	Do studies within the review compare presence/level of impact with non-carer samples?	Does the review identify evidence about impact for specific carer sub-groups relating to age, sex, socioeconomic status and geographical location?	Synthesis	Indicative risk of bias
Amer[29]	2019	19	1990-2017	Older people	'Caregiver burden', quality of life, perceived difficulty assisting recipient	No	No	Narrative	HIGH
Bom [30]	2019	15	2009-2017	Older people	Mental health, physical health	Yes, controls matched for caregiver health	Sex, SES, location (European region)	Narrative	HIGH
Ge [31]	2017	7	1999-2014	Older people with cancer	'Caregiver burden' prevalence and severity	No	Sex, SES, age	Narrative	MODERATE
Jansen [32]	2018	14	1994-2016	Older cancer survivors	Prevalence and predictors of 'caregiver burden', depression, anxiety, self-esteem, distress, communication issues, stress and QoL	Yes, general population	Sex, SES	Narrative	HIGH
Namasivayam [33]	2018	4	2002-2017	Older people	'Caregiver burden' associated with feeding difficulties	No	No	Narrative	HIGH
Ringer [34]	2017	9	1997-2012	Older people with frailty	'Caregiver burden', negative reactions to caregiving	Unclear	No	Narrative	MODERATE

For peer review only - http://bmjopen.bmj.com/site/about/guidelines.xhtml

Table 3. Overview of systematic reviews reporting evidence about carer interventions

Study	Date published	Number of studies in review	Data range of included studies	Population (care recipient)	Interventions evaluated	Outcome	Synthesis	Indicative risk of bias
Domingues[35]	2018	4	2013-2016	Older people with mild cognitive impairment	Cognitive, multicomponent	'Burden', quality of life, anxiety, mood	Narrative	HIGH
Guay[36]	2017	12	2000-2015	Older people	Internet-based interventions, including education, self-help therapeutic, and human-supported therapeutic.	Depression, anxiety, stress, 'strain', 'burden', physical burden, self-perceived health, quality of life	Narrative	HIGH
Lopez-Hartman[37]	2012	10	2002-2009	Older people	Individual and group psychosocial support	Depression, 'burden', stress, 'role strain', coping, knowledge, social support, anxiety, economic burden.	Narrative	HIGH
Mason[38]	2007	22	1985-2003	Older people	Respite	Satisfaction, 'burden'	Both	LOW
Murfield[39]	2019	4	2013-2018	Older people	Mindfulness + stress reduction, yoga and meditation.	Self-compassion, 'other health' outcome	Narrative	MODERATE
Shaw[40]	2009	104	2005-2009	Older people	Respite	'Burden', depression, anxiety, morale, anger, hostility, caregiving relationship	Both	LOW

For peer review only

Figure 1 here

Consequences of caring

Of the six systematic reviews reporting evidence about the consequences of caring for older people: none were judged to have a low risk of bias; two a moderate risk of bias;[32 35] and four a high risk of bias[30 31 33 34] (Supplementary materials table S2).

Moderate risk of bias

Two reviews reported that carers of older people experience 'burden'[a], depression and anxiety, but prevalence and severity were either not quantified, or estimates varied substantially.[32 35] For example, one review reported the prevalence of 'burden' among carers ranged from 1% to greater than 35%,[35] whilst the other reported estimates ranging from 37% to 100%.[32] The latter review presented limited evidence on which groups may be at greatest risk - this suggested that carers who are younger, male and with poor social and financial support may experience higher levels of 'burden'.[32] However, this evidence was not quantified in the review and so it is not possible to describe the difference in reported 'burden' between these groups.

High risk of bias

Four reviews reported that carers of older people experience 'burden', anxiety and stress, but prevalence and severity were either not quantified or highly variable.[30 31 33 34] Evidence about the severity of depression among carers was also variable.[30 31 33] For example, one review reported evidence of mild, moderate *and* severe depression, although it was not clear how 'mild', 'moderate' and 'severe' were defined.[30] Another review reported that the impact on depression varied, but with no supporting detail.[33] One review indicated that levels of carer stress, anxiety and

[a] Due to insufficient detail reported in publications about the outcomes 'carer burden' and 'role strain', we are unable to provide a clear definition of these outcomes.

For peer review only - http://bmjopen.bmj.com/site/about/guidelines.xhtml

distress were higher than those of the general population, although by how much was not reported.[33]

There was limited and mixed evidence about the consequences of caring for physical health in one review, with a positive impact on self-rated health but also evidence of increased pain and medication usage.[31] Evidence about which groups may be at greatest risk was reported in two reviews.[31 33] One suggested that the impact of caring on health was greater for females and married people.[31] Yet another reported mixed evidence about whether 'burden' was greater for male or female carers.[33]

Carer interventions

Of the six reviews reporting evidence about interventions to support those caring for older people, two were judged to have a low risk of bias;[39 41] one a moderate risk of bias;[40] and three a high risk of bias[36-38] (Supplementary materials table S3).

Low risk of bias

Two reviews reported evidence on respite interventions for carers of older people.[39 41] Carers valued respite, with high levels of reported satisfaction. Yet there was no strong evidence to suggest respite improved carers' mental health or 'carer burden.' There were gaps in the evidence about the impact of respite on many other types of outcomes (e.g. physical health).

Moderate rate of bias

Limited evidence was presented in one review, for the ability of a mindfulness stress reduction intervention to improve carer depression and anxiety. However, this intervention had no impact on stress, health service use, quality of life, or self-compassion. In the same study, a combined yoga and meditation intervention appeared to improve carer self-compassion and quality of life.[40]

High risk of bias

For peer review only - http://bmjopen.bmj.com/site/about/guidelines.xhtml

Reviews judged to have a high risk of bias reported evidence about cognitive-, psychosocial- and education-based interventions.

A review judged to be at high risk of bias, reported limited and inconsistent evidence that a cognitive-based intervention was beneficial to carers.[36] The components of this cognitive intervention included 'calendar training' and 'note taking', although the overall objective of this intervention was not clear.

Evidence for the impact of self-help and supported therapy on carer depression, anxiety and 'burden' was inconsistent.[37] Similarly, there was inconsistent evidence about the effect of individual psychosocial support interventions on 'carer burden' and depression.[38] Such individual psychosocial support interventions may improve carer stress and economic burden but this evidence was only identified in a single study in one review. There was no evidence of an effect on the outcome 'role strain'. Group psychosocial support interventions may improve carer stress, coping and knowledge but evidence was mixed for the outcomes of depression, 'carer burden' and 'role strain'.[38]

Finally, one review reported evidence about web-based educational interventions. Components of these educational interventions reported in the review included nurse and peer-led support to answer questions by email, provision of information on websites, and training in relaxation and exercise skills. There was no consistent evidence that these interventions were more beneficial than usual care.[37]

Key gaps in evidence about carer interventions

Supplementary materials table S4 maps intervention types against the outcomes reported in the reviews. Evidence for respite and psychosocial interventions was most wide ranging.[37-41] By contrast, there were notable gaps in evidence for cognitive, educational and multicomponent interventions. Outcomes reported less often were economic burden, relationships, and physical health.

Discussion

For peer review only - http://bmjopen.bmj.com/site/about/guidelines.xhtml

Older people's need for unpaid care is predicted to rise with population ageing.[2] Supporting the health and wellbeing of people providing unpaid care for older people, and mitigating the social and financial impacts of caring, should be a public health priority. Our rapid review of systematic reviews mapped current evidence to provide an overview of what is known, as well as identifying key gaps, about the health, social and financial impact of unpaid caring for older people, and how this group of carers can be best supported.

The challenges and demands of unpaid caring for older people are well documented.[10 11 13 14] However, the current evidence fails to fully quantify the consequences of caring for older people on carers' health and wellbeing. Clear measurement of outcomes, with comparison to the general population, is needed to allow the full extent of this impact to be understood. Furthermore, carers are not a homogenous population, and unpaid caring takes place in diverse circumstances. A greater focus on which groups of carers are most vulnerable to the impact of caring, and under which conditions, would further enhance our understanding of these issues.

The evidence reviewed here highlights gaps in our understanding and suggests that a wider consideration of relevant outcomes is needed. Research into the impact of caring and effectiveness of carer interventions must consider outcomes beyond mental health. In particular, physical health was largely neglected in these reviews about caring for older populations. A bias towards mental and psychological, rather than physical, health outcomes has also been noted by others in evidence about the consequences of caring.[42-44] Other reviews about the impact of caring at all ages report that carers are at increased risk of musculoskeletal conditions, cardiovascular disease, generalised cognitive deterioration and poor sleep.[42 45-47] Yet there is a clear gap in these outcomes in systematic reviews that focus on caring for older people. Similarly, we know that unpaid caring can lead to a loss of employment or a reduction in working hours.[48-50 51] Some carers face the demands of managing caring responsibilities alongside employment.[52] There are likely to be related impacts on financial and social wellbeing, but these outcomes were also absent in this

For peer review only - http://bmjopen.bmj.com/site/about/guidelines.xhtml

evidence. Future research must address the full breadth of potential consequences of being a carer, with consideration of these important but overlooked outcomes.

Another limitation of the current evidence base is the oft-used outcome 'carer burden'. This is an ambiguous and contentious term,[53] and input from our stakeholder group confirmed the unhelpful nature of this concept. We recommend that future evaluation and reviews avoid this outcome and focus on more meaningful and specific measures of impact.

A final limitation of the evidence relates to whether chosen outcomes are adequate to capture the likely impact of interventions. Poor choice of outcomes may underestimate or overlook the ways in which an intervention can effectively support and benefit carers of older people. This was most apparent in the reviews of respite interventions. Despite respite being valued by carers, there was no evidence that it improved carers' mental health or 'carer burden'. Yet respite alone cannot be expected to improve mental health, with other therapeutic support necessary to address these sorts of needs. There may also be outcomes where appropriate respite, even in the short-term, may show beneficial effects (e.g. stabilising carers' physical illness or injury). Evaluations could offer a more meaningful understanding of how an intervention can support carers by giving greater consideration to the outcomes measured and the potential pathways to impact.

Overall, our review of reviews indicates that a more robust and comprehensive evidence base is needed to inform policy and practice for supporting carers of older people. One mechanism to achieve this is by conceptualising unpaid caring as a social determinant of health. Social determinants are factors relating to life conditions and circumstances, such as age, income, and working conditions, that impact on health. To locate unpaid caring as a social determinant recognises that this responsibility is likely to place carers at greater risk of poor health. Conceptualising unpaid caring as a social determinant of health is possible with an adaption of Dahlgren and Whitehead's (1991) model (figure 2).[54] Working and living conditions are acknowledged as social determinants of health. Highlighting the similar role of

For peer review only - http://bmjopen.bmj.com/site/about/guidelines.xhtml

unpaid caring would be helpful here; caring responsibilities and their consequences shape health and are socially patterned. In an area of growing unmet need, framing unpaid caring as a social determinant of health may focus the attention of policymakers and drive the development of evidence-based interventions.

Figure 2 here

Moving forward, we propose the following future research priorities:

1. A high quality comprehensive systematic review about the impact of caring for older people on outcomes overlooked in current systematic reviews: physical health, and social and financial wellbeing. A systematic review of these outcomes should draw upon evidence from non-peer reviewed sources (for example, national statistical reports, third sector reports) and qualitative research to capture the full breadth of available evidence.

2. Clear quantification of the prevalence and severity of mental health outcomes and other illnesses experienced by carers of older people, with comparisons to the general population to highlight the extent of any impact on health and well-being, and potential inequalities between carers and non-carers

3. Robust development and evaluation of promising interventions for carers of older people, with consideration of pathways to impact and a broader range of relevant outcomes

The priorities outlined above have potential to address gaps in the current evidence base identified in our review, and to improve the overall quality and scope of evidence about caring for older people.

Strengths and limitations

Our chosen method was a review of published systematic reviews to avoid duplicating existing work. This is a valuable approach that has highlighted key gaps in the evidence base about caring for older people. A limitation of this method is that

For peer review only - http://bmjopen.bmj.com/site/about/guidelines.xhtml

primary studies, which have not yet been subjected to a systematic review, are inevitably excluded. However, nine of the 12 included systematic reviews were published within the last three years (2017-2019) and the remainder published between 2007 and 2012. The number of primary studies potentially excluded by using this method is therefore likely to be low.

Conclusion

Current evidence about caring for older people lacks the necessary detail to fully quantify the impact of unpaid caring on carers, and to identify how this population of carers should be supported. Classification of unpaid caring as a social determinant of health could be an effective lever to bring greater focus to this population in an area of growing unmet need. Further research is needed to identify suitable interventions, in order to support evidence-based policymaking and practice.

For peer review only

For peer review only - http://bmjopen.bmj.com/site/about/guidelines.xhtml

Funding statement

This paper presents independent research funded by Public Health England (NU-002487 RES/0290/0170). JL and BH are funded by the National Institute for Health Research (NIHR) Applied Research Collaboration North East and North Cumbria (ARC NENC, NIHR200173). The views expressed are those of the author(s) and not necessarily those of Public Health England or the NIHR.

Author contributions

GS developed the protocol, undertook all stages of the review and co-wrote the first draft of the paper; JL developed the protocol, undertook all stages of the review and co-wrote the first draft of the paper; PK developed the protocol, undertook all stages of the review and contributed to the final draft of the paper; OW developed the protocol, undertook record screening and data extraction for the review and contributed to the final draft of the paper; FB developed the protocol, undertook the study searches, and contributed to the final draft of the paper; DS developed the protocol, undertook record screening for the review and contributed to the final draft of the paper; CW developed the protocol, undertook record screening for the review and contributed to the final draft of the paper; SER developed the protocol, undertook record screening for the review and contributed to the final draft of the paper; DC planned the study, developed the protocol, undertook record screening for the review and co-wrote the first draft of the paper; BH planned the study, developed the protocol, undertook record screening and co-wrote the first draft of the paper.

Ethics statement
Not applicable: this study does not involve human participants.

Data sharing
Data sharing not applicable as no datasets generated for this study.

Competing interests
There are no competing interests.

References

1. Department of Social and Economic Affairs. World Population Ageing 2019 Highlights. *United Nations* 2019
2. Wittenburg R, Comas-Herrera A, Pickard L, et al. Future demand for long-term care in the UK. London: Joseph Rowntree Foundation, 2004.
3. Association LG. Adult Social Care Funding: 2016 State of the Nation Report: Local Government Association; 2016 [Available from: http://www.local.gov.uk/documents/10180/7632544/1+24+ASCF+state+of+the+nation+2016_WEB.pdf/e5943f2d-4dbd-41a8-b73e-da0c7209ec12 accessed 22/03/2017).
4. Commission CQ. The state of health and social care in England 2017/2018: Care Quality Commission, 2018.
5. Age UK. Age UK General Election Manifesto 2019, 2019.

For peer review only - http://bmjopen.bmj.com/site/about/guidelines.xhtml

6. Carers UK. Facts about carers, August 2019 Policy Briefing, 2019.
7. Carers UK. Key facts about carers and the people they care for 2015 [Available from: https://carers.org/key-facts-about-carers-and-people-they-care accessed 25.02.2020.
8. Carers UK. Missing out: the identification challenge. London: Carers UK, 2016.
9. Age UK. Older carers left to fill the gap as our social care system crumbles: Age UK, 2017.
10. Giraldo-Rodríguez L, Guevara-Jaramillo N, Agudelo-Botero M, et al. Qualitative exploration of the experiences of informal care-givers for dependent older adults in Mexico City. *Ageing and Society* 2018;39(11):2377-96. doi: 10.1017/s0144686x18000478
11. Saletti-Cuesta L, Tutton E, Langstaff D, et al. Understanding informal carers' experiences of caring for older people with a hip fracture: a systematic review of qualitative studies. *Disabil Rehabil* 2018;40(7):740-50. doi: 10.1080/09638288.2016.1262467 [published Online First: 2016/12/16]
12. Williams A, Sethi B, Duggleby W, et al. A Canadian qualitative study exploring the diversity of the experience of family caregivers of older adults with multiple chronic conditions using a social location perspective. *Int J Equity Health* 2016;15:40. doi: 10.1186/s12939-016-0328-6 [published Online First: 2016/03/05]
13. McGarry J, Arthur A. Informal caring in late life: a qualitative study of the experiences of older carers. *Journal of Advanced Nursing* 2008;33(2):182-89. doi: 10.1111/j.1365-2648.2001.01651.x
14. Rodger D, Neill MO, Nugent L. Informal carers' experiences of caring for older adults at home: a phenomenological study. *Br J Community Nurs* 2015;20(6):280-5. doi: 10.12968/bjcn.2015.20.6.280 [published Online First: 2015/06/05]
15. Carers UK, Age UK. Caring into later life: Carers UK, Age UK, 2015.
16. Carers UK. In Poor Health: Carers UK, 2004.
17. Al-Janabi H, Coast J, Flynn TN. What do people value when they provide unpaid care for an older person? A meta-ethnography with interview follow-up. *Soc Sci Med* 2008;67(1):111-21. doi: 10.1016/j.socscimed.2008.03.032 [published Online First: 2008/04/26]
18. Yeandle S. Caring for our carers: an international perspective on policy developments in the UK. *Juncture* 2016;23(1):57-62.
19. National Institute of Health & Care Excellence. Supporting adult carers, NICE Guideline 150 2020 [Available from: https://www.nice.org.uk/guidance/ng150 accessed 25.02.2020.
20. Dickinson C, Dow J, Gibson G, et al. Psychosocial intervention for carers of people with dementia: What components are most effective and when? A systematic review of systematic reviews. *International Psychogeriatrics* 2017;29(1):31-43.
21. Gilhooly KJ, Gilhooly ML, Sullivan MP, et al. A meta-review of stress, coping and interventions in dementia and dementia caregiving. *BMC Geriatr* 2016;16:106. doi: 10.1186/s12877-016-0280-8 [published Online First: 2016/05/20]
22. Huis In Het Veld JG, Verkaik R, Mistiaen P, et al. The effectiveness of interventions in supporting self-management of informal caregivers of people

For peer review only - http://bmjopen.bmj.com/site/about/guidelines.xhtml

with dementia; a systematic meta review. *BMC Geriatr* 2015;15:147. doi: 10.1186/s12877-015-0145-6 [published Online First: 2015/11/13]
23. Thomas S, Dalton J, Harden M, et al. Updated meta-review of evidence on support for carers. *NIHR Journals Library Health Services and Delivery Research* 2017;3:03.
24. Tricco AC, Antony J, Zarin W, et al. A scoping review of rapid review methods. *BMC Medicine* 2015;13(1):224. doi: 10.1186/s12916-015-0465-6
25. Haby MM, Chapman E, Clark R, et al. What are the best methodologies for rapid reviews of the research evidence for evidence-informed decision making in health policy and practice: a rapid review. *Health Res Policy Syst* 2016;14(1):83. doi: 10.1186/s12961-016-0155-7 [published Online First: 2016/11/26]
26. Canadian Agency for Drugs and Technologies in Health. Strings Attached: CADTH's Database Search Filters 2019 [Available from: https://www.cadth.ca/resources/finding-evidence/strings-attached-cadths-database-search-filters#syst accessed 28.02.2020.
27. Centre for Reviews and Dissemination (UK). Database of Abstracts of Reviews of Effects (DARE): Quality-assessed Reviews York (UK)1995 [Available from: https://www.ncbi.nlm.nih.gov/books/NBK285222/.
28. Ouzzani M, Hammady H, Fedorowicz Z, et al. Rayyan—a web and mobile app for systematic reviews. *Systematic Reviews* 2016;5(1):210. doi: 10.1186/s13643-016-0384-4
29. Whiting P, Savovic J, Higgins JP, et al. ROBIS: A new tool to assess risk of bias in systematic reviews was developed. *J Clin Epidemiol* 2016;69:225-34. doi: 10.1016/j.jclinepi.2015.06.005 [published Online First: 2015/06/21]
30. Amer Nordin A, Mohd Hairi F, Choo WY, et al. Care Recipient Multimorbidity and Health Impacts on Informal Caregivers: A Systematic Review. *Gerontologist* 2019;59(5):e611-e28. doi: 10.1093/geront/gny072 [published Online First: 2018/07/10]
31. Bom J, Bakx P, Schut F, et al. The Impact of Informal Caregiving for Older Adults on the Health of Various Types of Caregivers: A Systematic Review. *The Gerontologist* 2018;59(5):629-42.
32. Ge L, Mordiffi SZ. Factors Associated With Higher Caregiver Burden Among Family Caregivers of Elderly Cancer Patients: A Systematic Review. *Cancer nursing* 2017;40(6):471-78. doi: https://dx.doi.org/10.1097/NCC.0000000000000445
33. Jansen L, Dauphin S, van den Akker M, et al. Prevalence and predictors of psychosocial problems in informal caregivers of older cancer survivors - A systematic review: Still major gaps in current research. *European Journal of Cancer Care* 2018;27(6):1-13. doi: 10.1111/ecc.12899
34. Namasivayam-MacDonald AM, Shune SE. The Burden of Dysphagia on Family Caregivers of the Elderly: A Systematic Review. *Geriatrics (Basel)* 2018;3(2):10. doi: 10.3390/geriatrics3020030 [published Online First: 2019/04/24]
35. Ringer T, Hazzan AA, Agarwal A, et al. Relationship between family caregiver burden and physical frailty in older adults without dementia: a systematic review. *Syst Rev* 2017;6(1):55. doi: 10.1186/s13643-017-0447-1 [published Online First: 2017/03/16]

For peer review only - http://bmjopen.bmj.com/site/about/guidelines.xhtml

36. Domingues NS, Verreault P, Hudon C. Reducing Burden for Caregivers of Older Adults With Mild Cognitive Impairment: A Systematic Review. *Am J Alzheimers Dis Other Demen* 2018;33(7):401-14. doi: 10.1177/1533317518788151 [published Online First: 2018/07/26]
37. Guay C, Auger C, Demers L, et al. Components and Outcomes of Internet-Based Interventions for Caregivers of Older Adults: Systematic Review. *J Med Internet Res* 2017;19(9):e313. doi: 10.2196/jmir.7896 [published Online First: 2017/09/21]
38. Lopez-Hartmann M, Wens J, Verhoeven V, et al. The effect of caregiver support interventions for informal caregivers of community-dwelling frail elderly: a systematic review. *Int J Integr Care* 2012;12:e133. doi: 10.5334/ijic.845 [published Online First: 2013/04/18]
39. Mason A, Weatherly H, Spilsbury K, et al. The effectiveness and cost-effectiveness of respite for caregivers of frail older people. *J Am Geriatr Soc* 2007;55(2):290-9. doi: 10.1111/j.1532-5415.2006.01037.x [published Online First: 2007/02/17]
40. Murfield J, Moyle W, Jones C, et al. Self-Compassion, Health Outcomes, and Family Carers of Older Adults: An Integrative Review. *Clin Gerontol* 2019:1-14. doi: 10.1080/07317115.2018.1560383 [published Online First: 2019/01/08]
41. Shaw C, McNamara R, Abrams K, et al. Systematic review of respite care in the frail elderly. *Health Technol Assess* 2009;13(20):1-224, iii. doi: 10.3310/hta13200 [published Online First: 2009/04/28]
42. Cottagiri S, Sykes P. Key health impacts and support systems for informal carers in the UK: A thematic review *Journal of Health and Social Sciences* 2019 doi: doi10.19204/2019/kyhl11
43. Pinquart M, Sörensen S. Correlates of Physical Health of Informal Caregivers: A Meta-Analysis. *The Journals of Gerontology: Series B* 2007;62(2):P126-P37. doi: 10.1093/geronb/62.2.P126
44. Clancy RL, Fisher GG, Daigle KL, et al. Eldercare and Work Among Informal Caregivers: A Multidisciplinary Review and Recommendations for Future Research. *Journal of Business and Psychology* 2020;35(1):9-27. doi: 10.1007/s10869-018-9612-3
45. Fonareva I, Oken BS. Physiological and functional consequences of caregiving for relatives with dementia. *Int Psychogeriatr* 2014;26(5):725-47. doi: 10.1017/s1041610214000039 [published Online First: 2014/02/11]
46. Romero-Martínez Á, Hidalgo-Moreno G, Moya-Albiol L. Neuropsychological consequences of chronic stress: the case of informal caregivers. *Aging & Mental Health* 2020;24(2):259-71. doi: 10.1080/13607863.2018.1537360
47. Vitaliano PP, Zhang J, Scanlan JM. Is caregiving hazardous to one's physical health? A meta-analysis. *Psychological bulletin* 2003;129(6):946-72. doi: 10.1037/0033-2909.129.6.946 [published Online First: 2003/11/06]
48. Carers UK. In sickness and in health, 2012.
49. King D, Pickard L. When is a carer's employment at risk? Longitudinal analysis of unpaid care and employment in midlife in England. *Health Soc Care Community* 2013;21(3):303-14. doi: 10.1111/hsc.12018 [published Online First: 2013/01/30]

For peer review only - http://bmjopen.bmj.com/site/about/guidelines.xhtml

50. Van Houtven CH, Coe NB, Skira MM. The effect of informal care on work and wages. *Journal of Health Economics* 2013;32(1):240-52. doi: https://doi.org/10.1016/j.jhealeco.2012.10.006
51. Carers UK. Juggling work and unpaid care, 2019.
52. Vlachantoni A. The demographic characteristics and economic activity patterns of carers over 50: Evidence from the English Longitudinal Study of Ageing. *Population trends* 2010;141(141):51-73.
53. Bastawrous M. Caregiver burden--a critical discussion. *Int J Nurs Stud* 2013;50(3):431-41. doi: 10.1016/j.ijnurstu.2012.10.005 [published Online First: 2012/11/08]
54. Dahlgren G, Whitehead M. European strategies for tackling social inequities in health: Levelling up Part 2. *WHO Collaborating Centre for Policy Research on Social Determinants of Health, University of Liverpool* 1991

Figure legends

Figure 1. PRISMA Flowchart

Figure 2.Unpaid caring as a social determinant of health: an adaptation of Dahlgren & Whitehead's (1991) model

For peer review only

For peer review only - http://bmjopen.bmj.com/site/about/guidelines.xhtml

Figure 1.PRISMA flowchart of study selection

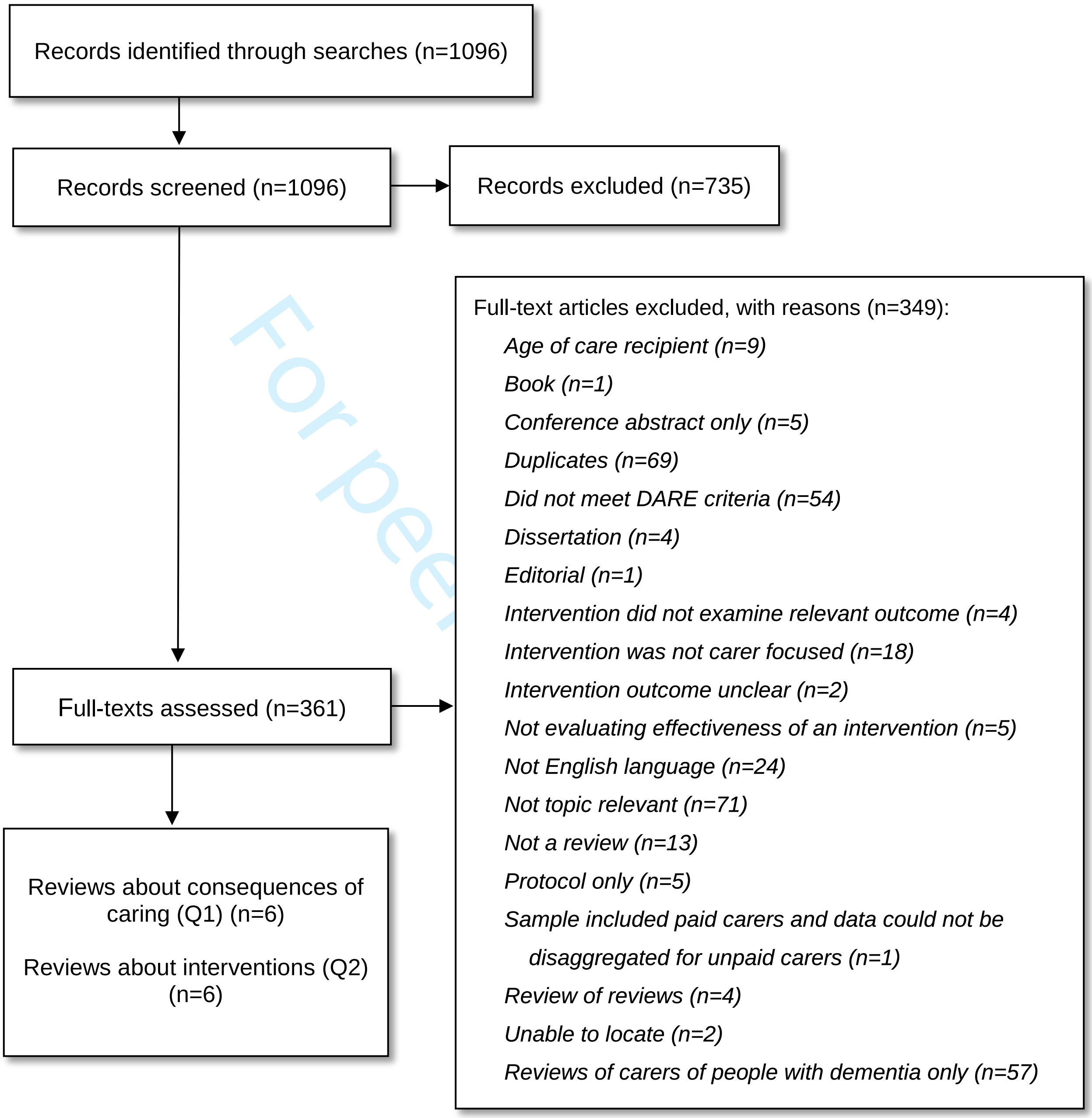

For peer review only - http://bmjopen.bmj.com/site/about/guidelines.xhtml

Figure 2. Unpaid caring as a social determinant of health: an adaptation of Dalgren & Whitehead's (1991) model

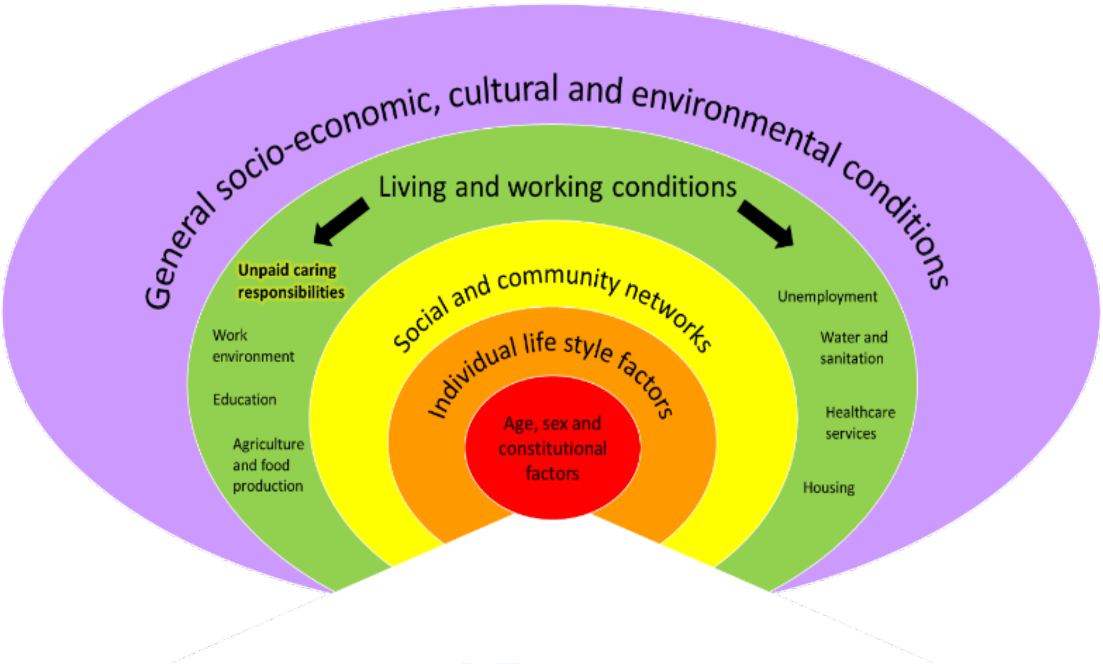

For peer review only - http://bmjopen.bmj.com/site/about/guidelines.xhtml

For peer review only

For peer review only - http://bmjopen.bmj.com/site/about/guidelines.xhtml

Supplementary materials

Appendix A: Search strategy as applied to MEDLINE

Database(s): **Ovid MEDLINE(R)** 1946 to January Week 2 2020; Searched 21st January

#	Searches	Results
1	meta-analysis.pt.	109635
2	meta-analysis/ or systematic review/ or meta-analysis as topic/ or "meta analysis (topic)"/ or "systematic review (topic)"/ or exp technology assessment, biomedical/	205585
3	((systematic* adj3 (review* or overview* or analys*)) or (methodologic* adj3 (review* or overview* or analys*))).ti,ab,kf,kw.	150031
4	(((quantitative or narrative) adj3 (review* or overview* or synthes*)) or (research adj3 (integrati* or overview*))).ti,ab,kf,kw.	17348
5	((integrative adj3 (review* or overview*)) or (collaborative adj3 (review* or overview*)) or (pool* adj3 analy*)).ti,ab,kf,kw.	20727
6	(data synthes* or data extraction* or data abstraction*).ti,ab,kf,kw.	20899
7	(handsearch* or hand search*).ti,ab,kf,kw.	7850
8	(mantel haenszel or peto or der simonian or dersimonian or fixed effect* or latin square*).ti,ab,kf,kw.	21652
9	(met analy* or metanaly* or technology assessment* or HTA or HTAs or technology overview* or technology appraisal*).ti,ab,kf,kw.	7271
10	(meta regression* or metaregression*).ti,ab,kf,kw.	6630
11	(meta-analy* or metaanaly* or systematic review* or biomedical technology assessment* or bio-medical technology assessment*).mp,hw.	235567
12	(medline or cochrane or pubmed or medlars or embase or cinahl).ti,ab,hw.	166667
13	(cochrane or (health adj2 technology assessment) or evidence report).jw.	18482
14	(comparative adj3 (efficacy or effectiveness)).ti,ab,kf,kw.	10523
15	(outcomes research or relative effectiveness).ti,ab,kf,kw.	7615
16	((indirect or indirect treatment or mixed-treatment) adj comparison*).ti,ab,kf,kw.	1649
17	((meta-narrative or meta-ethnograph* or mixed method* or critical or thematic or realist or framework) adj3 (review* or synthes*)).ti,ab,kf,kw.	20949
18	or/1-17	395639
19	((carer* or caregiver* or caring or care partner* or "family care" or "spousal care" or "unpaid care" or "informal care") adj5 (older or senior* or elder* or dement* or alzheimer*)).ti,ab.	9395
20	18 and 19	613

For peer review only

Table S1. Abbreviated risk of bias criteria, adapted from the ROBIS tool[1]

1. Can searches be considered exhaustive and thus prone to minimal bias?
2. Are review criteria clear, and thus prone to minimal selection bias?
3. Is the quality assessment incorporated into the synthesis to prioritise more robust evidence, or considered in relation to the conclusions drawn?
4. Is the synthesis transparent and clear: sufficient study details are reported; synthesised evidence is referenced appropriately?
5. Do review findings and synthesis avoid emphasising results based only on statistical significance?

For peer review only

Table S2. The consequences of caring for older populations: summary of evidence by review risk of bias

Study	Type of consequence/impact reported: Health (physical and mental)	Social	Finance	Quality of life	Other	Evidence about which groups may be at greater risk
	Evidence from reviews with a moderate risk of bias					
Ge [31]	'BURDEN' (PREVALENCE)<br><br>Across 3 studies, the proportion of carers reporting levels of 'carer burden' varied from 37% to 100%.<br><br>'BURDEN' (SEVERITY)<br><br>The percentage of carers who reported high 'burden' ranged from 1% to greater than 35% (across 4 studies).	NA	NA	NA	NA	SEX<br><br>Limited evidence of an association between sex of carer (male) and higher 'carer burden'.<br><br>AGE<br><br>Younger carer age was associated with higher 'carer burden'.<br><br>SES<br><br>Limited evidence that lack of social support and insufficient financial support were factors contributing to higher 'carer burden'.
Ringer[34]	'BURDEN', DEPRESSION, ANXIETY<br><br>Carers of frail older adults experienced 'burden', depression and anxiety, but	NA	NA	NA	NA	None

For peer review only

	it was not possible to quantify this from included studies. Evidence from one included study indicated that the 'burden' experienced by carers of frail older people was less than that of carers of those with cancer, diabetes and dementia.					
	Evidence from reviews with a high risk of bias					
Amer [29]	DEPRESSION (SEVERITY)<br><br>Evidence about the level of depression experienced by carers differed across studies; levels were judged to be both mild to moderate, and severe, although it is not clear how these were defined.<br><br>'BURDEN' (SEVERITY)<br><br>'Carer burden' was judged to be low to moderate, although it is not clear how these are defined.	NA	NA	Carers' quality of life was lower compared to national levels.	Limited findings suggest an association between carers' perceived difficulty in assisting care recipients and differences in cultural values.	None
Bom [30]	DEPRESSION (PREVALENCE)<br><br>Caring was associated with higher prevalence of depressive feelings and lower mental health scores.<br><br>PHYSICAL HEALTH	NA	NA	NA	NA	SEX<br><br>Impact of caring on health is greater for females and those married.

For peer review only

	There was mixed evidence about impact on physical health; review authors suggest this reflects different outcome measures.				
Jansen [32]	DISTRESS AND ANXIETY (SEVERITY)<br><br>There was limited evidence showing greater distress and anxiety in carers of older cancer survivors compared to the general population.<br><br>SELF-ESTEEM<br><br>There was limited evidence that caring was associated with a high self-esteem.<br><br>DEPRESSION<br><br>Evidence of impact on depression was reported to be varied (although full details are not reported). Authors suggest that this was due to the variety of instruments used to measure the outcome.<br><br>'BURDEN' (PREVALENCE)		Limited evidence shows an association between lower quality of life and carers of older cancer survivors compared with the general population	There was limited evidence that carers reported difficulties in talking to the care recipients about their illness, suggesting communication issues	SEX<br><br>One study reported that being a female carer was a predictor for 'burden' while another study found that being a male spousal carer was a risk factor for 'burden'.

For peer review only

	Limited evidence indicated 40% of carers experienced high 'carer burden'.<br><br>STRESS (SEVERITY)<br><br>Limited evidence that carers experience higher levels of stress than the UK general adult population

For peer review only

Namasivayam [33]	'BURDEN' (SEVERITY)<br><br>Dysphagia in older care recipients is associated with increased 'carer burden'.	NA	NA	NA	NA

For peer review only

Table **S3. Interventions for carers: summary of evidence by review risk of bias**

Study	Interventions for carers of older people	
	**Findings**	**Sub-group analysis**
	Evidence from reviews with a low risk of bias	
Shaw [40]	Pooled analyses indicated respite care had no effect on 'carer burden' and anxiety, and little effect on carer depression. Some evidence indicated that respite was associated with worse carer quality of life, although the review authors note there were potential sources of bias in these studies. Other evidence indicated respite may improve the carer-recipient relationship. Home help-based respite may improve carers' morale.	None reported.
Mason [38]	There was no consistent evidence to indicate respite care was beneficial to carers. Pooled analysis indicated no effect on 'carer burden', and a small improvement in depression, although authors note a methodological flaw which undermines the reliability of this finding. However, satisfaction levels were generally high for all types of respite compared to usual care.	*Although the authors report findings from one study about outcomes for sub-groups, it is not clear if this refers to the carer or recipient. Other sub-group findings reported relate to likelihood of the carer 'institutionalising' the care recipient.*
	Evidence from reviews with a moderate risk of bias	
Murfield [39]	There was limited evidence (1 study) that a mindfulness stress reduction intervention may improve carer depression and anxiety but not other outcomes. There was limited evidence (1 study) that a yoga + meditation based intervention improved carer self-compassion and quality of life.	None reported.
	Evidence from reviews with a high risk of bias	
Domingues [35a]	There was limited and mixed evidence for a cognitive intervention and a multicomponent intervention.	None reported.
Guay [36]	There was no consistent evidence to indicate web-based education interventions were beneficial to carers, and mixed evidence for therapy interventions.	None reported.
Lopez-Hartman [37]	There was inconsistent evidence about whether individual and group psychosocial support interventions are beneficial for carers, with improvements to some outcomes but not others.	One study reported that carers sharing a household with care recipients may benefit more from an individual psychosocial support group compared to those living apart.

[a]*Other interventions were reported in this review but did not target only the carer and were primarily oriented towards supporting the care recipient.*

For peer review only

Table S4. Reviews reporting outcomes by intervention classification

	INTERVENTION TYPE				
**OUTCOME**	**Respite**	**Psychosocial**	**Cognitive**	**Education**	**Multicomponent**
**'Burden'**	Shaw 2009<br>Mason 2007	Lopez-Hartman 2012<br>Guay 2017	Domingues 2018	Guay 2017	Domingues 2018
**Depression/mood**	Shaw 2009	Lopez-Hartman 2012<br>Guay 2017	Domingues 2018	Guay 2017	Domingues 2018
**Anxiety**	Shaw 2009	Lopez-Hartman 2012<br>Guay 2017	-	-	-
**Stress, wellbeing, quality of life, 'role strain', morale, self-compassion**	Shaw 2009	Lopez-Hartman 2012<br>Murfield 2019	-	Guay 2017	
**Health, physical burden, 'other' health**	-	Guay 2017	-	-	-
**Anger, hostility**	Shaw 2009	-	-	-	-
**Knowledge**	-	Lopez-Hartman 2012<br>Guay 2017	-	-	-
**Coping**	-	Lopez-Hartman 2012<br>Guay 2017	-	-	-
**Relationships**	Shaw 2009	-	-	-	-
**Satisfaction**	Mason 2007	-	-	-	-
**Economic burden**	-	Lopez-Hartman 2012	-	-	-

For peer review only

PRISMA 2009 Checklist

Section/topic	#	Checklist item	Reported on page #
**TITLE**			
Title	1	Identify the report as a systematic review, meta-analysis, or both.	1
**ABSTRACT**			
Structured summary	2	Provide a structured summary including, as applicable: background; objectives; data sources; study eligibility criteria, participants, and interventions; study appraisal and synthesis methods; results; limitations; conclusions and implications of key findings; systematic review registration number.	3
**INTRODUCTION**			
Rationale	3	Describe the rationale for the review in the context of what is already known.	5-6
Objectives	4	Provide an explicit statement of questions being addressed with reference to participants, interventions, comparisons, outcomes, and study design (PICOS).	6
**METHODS**			
Protocol and registration	5	Indicate if a review protocol exists, if and where it can be accessed (e.g., Web address), and, if available, provide registration information including registration number.	NA
Eligibility criteria	6	Specify study characteristics (e.g., PICOS, length of follow-up) and report characteristics (e.g., years considered, language, publication status) used as criteria for eligibility, giving rationale.	7-8, Table 1
Information sources	7	Describe all information sources (e.g., databases with dates of coverage, contact with study authors to identify additional studies) in the search and date last searched.	8
Search	8	Present full electronic search strategy for at least one database, including any limits used, such that it could be repeated.	Supplementary materials
Study selection	9	State the process for selecting studies (i.e., screening, eligibility, included in systematic review, and, if applicable, included in the meta-analysis).	8
Data collection process	10	Describe method of data extraction from reports (e.g., piloted forms, independently, in duplicate) and any processes for obtaining and confirming data from investigators.	8-9
Data items	11	List and define all variables for which data were sought (e.g., PICOS, funding sources) and any assumptions and simplifications made.	8-9
Risk of bias in individual studies	12	Describe methods used for assessing risk of bias of individual studies (including specification of whether this was done at the study or outcome level), and how this information is to be used in any data synthesis.	8-9
Summary measures	13	State the principal summary measures (e.g., risk ratio, difference in means).	NA
Synthesis of results	14	Describe the methods of handling data and combining results of studies, if done, including measures of consistency (e.g., I^2) for each meta-analysis.	9

For peer review only

PRISMA 2009 Checklist

Section/topic	#	Checklist item	Reported on page #
Risk of bias across studies	15	Specify any assessment of risk of bias that may affect the cumulative evidence (e.g., publication bias, selective reporting within studies).	NA
Additional analyses	16	Describe methods of additional analyses (e.g., sensitivity or subgroup analyses, meta-regression), if done, indicating which were pre-specified.	NA
**RESULTS**			
Study selection	17	Give numbers of studies screened, assessed for eligibility, and included in the review, with reasons for exclusions at each stage, ideally with a flow diagram.	9, and figure 1
Study characteristics	18	For each study, present characteristics for which data were extracted (e.g., study size, PICOS, follow-up period) and provide the citations.	9, tables 2 and 3
Risk of bias within studies	19	Present data on risk of bias of each study and, if available, any outcome level assessment (see item 12).	10-11, supplementary materials table S2 and S3
Results of individual studies	20	For all outcomes considered (benefits or harms), present, for each study: (a) simple summary data for each intervention group (b) effect estimates and confidence intervals, ideally with a forest plot.	Supplementary materials tables S2 and S3
Synthesis of results	21	Present results of each meta-analysis done, including confidence intervals and measures of consistency.	NA – no meta analysis
Risk of bias across studies	22	Present results of any assessment of risk of bias across studies (see Item 15).	10-11, supplementary materials table S2 and S3
Additional analysis	23	Give results of additional analyses, if done (e.g., sensitivity or subgroup analyses, meta-regression [see Item 16]).	NA
**DISCUSSION**			
Summary of evidence	24	Summarize the main findings including the strength of evidence for each main outcome; consider their relevance to key groups (e.g., healthcare providers, users, and policy makers).	12-15
Limitations	25	Discuss limitations at study and outcome level (e.g., risk of bias), and at review-level (e.g., incomplete retrieval of identified research, reporting bias).	15

For peer review only

For peer review only - http://bmjopen.bmj.com/site/about/guidelines.xhtml

PRISMA 2009 Checklist

Conclusions	26	Provide a general interpretation of the results in the context of other evidence, and implications for future research.	12-15
**FUNDING**			
Funding	27	Describe sources of funding for the systematic review and other support (e.g., supply of data); role of funders for the systematic review.	Title page

From: Moher D, Liberati A, Tetzlaff J, Altman DG, The PRISMA Group (2009). Preferred Reporting Items for Systematic Reviews and Meta-Analyses: The PRISMA Statement. PLoS Med 6(7): e1000097. doi:10.1371/journal.pmed1000097

For more information, visit: **www.prisma-statement.org**.

For peer review only

For peer review only - http://bmjopen.bmj.com/site/about/guidelines.xhtml
